# Effects of Novel versus Conventional Porcelain Surface Treatments on Shear Bond Strength of Orthodontic Brackets: A Systematic Review and Meta-Analysis

**DOI:** 10.1155/2022/8246980

**Published:** 2022-01-06

**Authors:** Farhad Sobouti, Mehdi Aryana, Sepideh Dadgar, Reza Alizadeh Navaei, Vahid Rakhshan

**Affiliations:** ^1^Dental Research Center, Mazandaran University of Medical Sciences, Sari, Iran; ^2^Orthodontic Department, Faculty of Dentistry, Mazandaran University of Medical Sciences, Sari, Iran; ^3^Student Research Committee, Faculty of Dentistry, Mazandaran University of Medical Sciences, Sari, Iran; ^4^Gastrointestinal Cancer Research Center, Non-Communicable Diseases Institute, Mazandaran University of Medical Sciences, Sari, Iran; ^5^Department of Anatomy, Dental School, Azad University of Medical Sciences, Tehran, Iran

## Abstract

**Background:**

Despite the importance of identifying proper novel porcelain preparation techniques to improve bonding of orthodontic brackets to porcelain surfaces, and despite the highly controversial results on this subject, no systematic review or meta-analysis exists in this regard.

**Objective:**

To comparatively summarize the effects of all the available porcelain surface treatments on the shear bond strength (SBS) and adhesive remnant index (ARI) of orthodontic brackets (metal, ceramic, polycarbonate) bonded to feldspathic porcelain restorations. *Search Methods*. A search was conducted for articles published between January 1990 and February 2021 in PubMed, MeSH, Scopus, Web of Science, Cochrane, Google Scholar, and reference lists. *Eligibility Criteria*. English-language articles comparing SBS of feldspathic porcelain's surface preparation methods for metal/ceramic/polycarbonate orthodontic brackets were included. Articles comparing silanes/bonding agents/primers without assessing roughening techniques were excluded. *Data Analysis*. Studies were summarized and risk of bias assessed. Each treatment's SBS was compared with the 6 and 10 MPa recommended thresholds. Studies including comparator (HF [hydrofluoric acid] + silane + bonding) were candidates for meta-analysis. ARI scores were dichotomized. Fixed- and random-effects models were used and forest plots drawn. Egger regressions and/or funnel plots were used to assess publication biases.

**Results:**

Thirty-two studies were included (140 groups of SBS, 82 groups of ARI). Bond strengths of 21 studies were meta-analyzed (64 comparisons in 14 meta-analyses). ARIs of 12 articles were meta-analyzed (28 comparisons in 8 meta-analyses). Certain protocols provided bond strengths poorer than HF + silane + bonding: “abrasion + bonding, diamond bur + bonding, HF + bonding, Nd:YAG laser (1 W) + silane + bonding, CO_2_ laser (2 W/2 Hz) + silane + bonding, and phosphoric acid + silane + bonding.” Abrasion + HF + silane + bonding might act almost better than HF + silane + bonding. Abrasion + silane + bonding yields controversial results, being slightly (marginally significantly) better than HF + silane + bonding. Some protocols had controversial results with their overall effects being close to HF + silane + bonding: “Cojet + silane + bonding, diamond bur + silane + bonding, Er:YAG laser (1.6 W/20 Hz) + silane + bonding.” Few methods provided bond strengths similar to HF + silane + bonding without much controversy: “Nd:YAG laser (2 W) + silane + bonding” and “phosphoric acid + silane + bonding” (in ceramic brackets). ARIs were either similar to HF + silane + bonding or relatively skewed towards the “no resin on porcelain” end. The risk of bias was rather low. *Limitations*. All the found studies were in vitro and thus not easily translatable to clinical conditions. Many metasamples were small.

**Conclusions:**

The preparation methods HF + silane + bonding, abrasion + HF + silane + bonding, Nd:YAG (2 W) + silane + bonding, and phosphoric acid + silane + bonding (in ceramic brackets) might provide stronger bonds.

## 1. Introduction

Orthodontic brackets should tolerate masticatory forces, by proper adhesion to the tooth, which is simulated in vitro by shear bond strength (SBS) [1]. The bond of the orthodontic brackets to the tooth surface or restoration must be strong enough to withstand the forces of orthodontic treatment and masticatory movements without displacement or failure [2]. At the same time, it should not be excessively strong that it damages the dental or restoration surface while removing the brackets at the end of orthodontic treatment [2].

Nowadays, beauty is an important factor in orthodontic treatments [3]; hence, the number of adults seeking orthodontic treatment almost doubled from 2010 to 2014 (from 14% to 27%) [4, 5]. Many adult orthodontic patients have a resin, amalgam, gold, acrylic resin, or porcelain restoration in their mouth [6].

Compared to the enamel surface, the bond of orthodontic brackets to ceramic surfaces might be associated with more failure [7]. Optimal adhesion of the bracket to the porcelain surface is a concern in orthodontics, because the bond should sustain orthodontic forces without jeopardizing porcelain integrity after debonding [2, 8]. Glazed feldspathic porcelain is not a suitable surface for orthodontic resin and bonding penetration due to the physical properties of the glazed surfaces and the chemical properties of the bonding resins [9]. Therefore, to bond brackets to the surface of glazed porcelain, a multistep process must be performed, including porcelain deglazing [10] or sandblasting [11], precise isolation, surface preparation, rinsing, drying, and finally bonding [7–9, 11–15].

Chemical, physical, and mechanical methods such as orthophosphoric acid, maleic acid, air abrasion, and laser have been used to prepare the enamel surface for orthodontic brackets [16–21]. But the procedures of preparing the surface of ceramic restorations are not exactly the same as enamel. Different approaches such as hydrofluoric acid, orthophosphoric acid, maleic acid, Monobond Etch & Prime, ceramic primer, laser, burs and air abrasion are used to bond orthodontic brackets to porcelain surface [2, 6, 12, 22–39]. However, many results are controversial and would benefit from aggregation and meta-analysis.

Porcelain has been used in cosmetic dentistry for many years due to its special physical properties such as strength and elegance. This material is brittle due to low tensile strength and high compressive strength. Dental porcelains are divided into several categories based on the phase ratio of crystalline to glass, including feldspathic porcelains, aluminous porcelains, or glass ceramics. Of these, feldspathic porcelains have many applications in ceramic fused-to-metal restorations as well as their ability to make strong and attractive restorations [12, 34, 40]. Since feldspathic porcelain restorations are common; bracket bonding to these surfaces is of importance to orthodontists. The protocols suggested and tested for improving shear bond strength of orthodontic brackets bonded to feldspathic porcelains (without increasing the SBS to excessively high and unsafe levels) are largely diverse and controversial. And there is no systematic review or meta-analysis on this subject. Therefore, this systematic review and meta-analysis was conducted to comparatively summarize the available methodologies in the literature, with emphasis on surface preparation techniques, and to highlight more effective treatments. The objectives were (1) to evaluate and summarize the available literature on SBS and ARI of brackets bonded to feldspathic porcelain surface, (2) to compare various techniques of surface preparation with the gold standard [41] in terms of their bond strengths and ARIs (as meta-analyses), and (3) to compare the mean SBS of each group of each study with the bond strengths recommended for orthodontic brackets in vitro [1].

## 2. Materials and Methods

### 2.1. Information Sources and Search Strategy

The review protocol was not registered beforehand. To find and compare (in vivo or in vitro) studies on porcelain surface preparation methods for orthodontic bracket bonding, English-language articles published from January 1, 1990, to February 26, 2021, were searched by at least two authors in the PubMed, Scopus, Web of Science, and Cochrane Library databases. Related terms were searched in the Medical Subject Headings (MeSH) database, and finally, the terms “Orthodontic Bracket” AND “Porcelain” AND “Shear Bond Strength” were selected as the main search keywords. Moreover, manual search was done in Google Scholar and also in reference lists of found full articles. Gray literature as well was searched.

### 2.2. Inclusion and Exclusion Criteria

All English-language (in vivo or in vitro) articles comparing porcelain surface preparation methods for orthodontic brackets were included in the study. According to the population, intervention, comparison, and outcomes (PICO) criteria, the desired population was considered in vitro studies assessing the SBS (and ARI) of orthodontic brackets to feldspathic porcelain surfaces. Interventions were the surface treatments which were considered in each study according to the surface preparation methods (including surface roughening technique, use or omission of silane, use of bonding). The comparator was the HF + silane + bonding treatment, as the gold standard [41]. The outcomes were the average value and standard deviation reported for shear bond strength and frequencies for adhesive remnant index scores. These outcomes limited the search results to in vitro studies only, as no in vivo studies had assessed SBS or ARI.

The following articles were excluded: reviews, case reports, editorials, guidelines, letter to the editors, and abstracts from conferences; articles not written in English; duplicate articles; articles with no available full text; articles which did not compare different surface preparation methods; articles which did not work on “feldspathic” porcelain; merely comparing different types of silanes or bonding agents or primers without assessing roughening techniques; and articles which did not measure “shear bond strength” or “adhesive remnant index.”

### 2.3. Data Items

The data were collected by at least two authors. To be included in the quantitative part (the meta-analysis) regarding any given surface treatment, at least two different studies with that particular surface treatment needed to exist. As mentioned above, the outcomes of interest were the average value and standard deviation reported for shear bond strength and frequencies for adhesive remnant index scores. Moreover, studies needed to include the HF + silane + bonding treatment, as the gold standard [41]. This group would be treated as the control group, in comparison with other treatments. Therefore, articles in which HF + silane were not applied were excluded from the quantitative meta-analyses. There were some differences in the definitions of ARI scores in some studies: a definition was a 4-scale score of zero to 3 (0 = no adhesive remnant on the porcelain surface, 1 = less than 50% remnant on the porcelain surface, 2 = more than 50%, and 3 = 100% remnant on the porcelain). Another definition was a 5-scale score of 5 to 1 (5 = no adhesive remnant on the porcelain surface, 4 = less than 10% remnant on the porcelain, 3 = between 10% and 90% remnant on the porcelain, 2 = more than 90% remnant, and 1 = 100% adhesive remaining on the porcelain). All results that were compatible with each outcome domain in each study were searched for.

The other variables for which data were sought were the country of research, sample size, number of groups, bracket types, surface roughening methods, silane application protocols, primer application protocols, bonding application protocols, thermocycling, SBS crosshead speed, and types of ARI grouping. The bracket types included were metal and ceramic brackets. One study with polycarbonate brackets was excluded.

The grouping was done firstly based on the type of brackets in use into metal, ceramic, and polycarbonate brackets. Next, in each bracket group, there were groups with or without silane application (all groups employed bonding agents except a few cases). Afterwards, these groups were divided further according to the surface treatment techniques, being one of the following: HF (hydrofluoric acid), PA (phosphoric acid), abrasion, silica coating, diamond bur, Er:YAG laser, Nd:YAG laser, Er:CrYSGG laser, CO_2_ laser, Ti:sapphire femtosecond laser, and femtosecond laser. The HF and phosphoric acid groups, despite using different concentrations (HF concentration was mainly 9.6% or about 10% and for PA was mostly 37%), were not separated to avoid excessive dispersion of the metasamples. Laser groups were divided into different groups based on the power and frequency used. Abrasion groups, despite the use of particles of different sizes (mostly 25 or 50 microns), were not separated to prevent overdispersion of the metasamples. The diamond bur groups, despite using different burs (mostly not mentioned, but some said fine or extra-fine burs), were not separated to prevent overdispersion of the metasamples. Polycarbonate brackets were not included in the quantitative meta-analysis, as there was only one study in this category.

Meta-analyses were performed in a separate metal bracket category and in a separate ceramic bracket category. In each bracket category, HF + silane (+bonding) preparation method was considered as the gold standard. In each of the metal and ceramic categories, studies that did not have this group were not included in the meta-analysis. Among the various surface preparation groups, those entered into the meta-analysis that contained more than one study in that group. For example, the abrasion + silane + bonding group was used in 14 studies (that also had the basic precondition, i.e., having the HF + silane + bonding group). But for example, the CO_2_ laser group (2 W, 2 Hz) + silane + bonding was not included in the meta-analysis, because there was no study that had the initial precondition, or there was only one study that had the initial precondition.

Overall, the following groups were found (the ones marked with an asterisk were included in the quantitative meta-analyses of SBS as well):

*Metal brackets*: HF + silane + bonding∗, HF + bonding (no silane)∗, HF + Er:CrYSGG laser (3 W, 10 Hz) + bonding (no silane), HF + Er:YAG laser (3 W, 10 Hz) + bonding (no silane), phosphoric acid 37% + bonding (no silane)∗, phosphoric acid 37% + silane + bonding∗, abrasion + bonding (no silane)∗, abrasion + silane + bonding∗, abrasion + HF + bonding (no silane), abrasion + HF + silane + bonding∗, Cojet (a type of silica coating) + silane + bonding∗, diamond bur + bonding (no silane)∗, diamond bur + silane + bonding∗, Er:YAG laser (1.6 W, 20 Hz) + silane + bonding∗, Er:YAG laser (2 W, 10 Hz) + silane + bonding, Er:YAG laser (2 W, 20 Hz) + silane + bonding, Er:YAG laser (2 W, 10 Hz) + bonding (no silane), Er:YAG laser (3 W) + bonding (no silane), Er:YAG laser (3.2 W, 20 Hz) + silane + bonding, Er:CrYSGG laser (3 W, 10 Hz) + bonding (no silane), Er:YAG laser (3 W, 10 Hz) + bonding (no silane), Nd:YAG laser (0.75 W) + silane + bonding, Nd:YAG laser (0.8 W) + bonding (no silane), Nd:YAG laser (1 W) + silane + bonding∗, Nd:YAG laser (1.25 W) + silane + bonding, Nd:YAG laser (1.5 W) + silane + bonding, Nd:YAG laser (2 W, 10 Hz) + silane + bonding, Nd:YAG laser (2 W, 20 Hz) + silane + bonding, Nd:YAG laser (3 W, 20 Hz) + silane + bonding, Nd:YAG laser (4 W, 40 Hz) + silane + bonding, Ti:sapphire femtosecond laser (0.45 W, 1 kHz) + silane + bonding, femtosecond laser (0.75 W, 1 kHz) + silane + bonding, CO_2_ laser (2 W, 2 Hz) + bonding (no silane), CO_2_ laser (2 W, 2 Hz) + silane + bonding, CO_2_ laser (10 W, 200 Hz) + silane + bonding, CO_2_ laser (15 W, 200 Hz) + silane + bonding, CO_2_ laser (20 W, 200 Hz) + silane + bonding.

*Ceramic brackets*: HF + silane + bonding∗, HF + bonding (no silane), phosphoric acid 37% + silane + bonding∗, abrasion + silane + bonding, abrasion + HF + silane + bonding, diamond bur + silane + bonding, diamond bur + phosphoric acid 37% + bonding (no silane), Nd:YAG laser (15 Hz) + silane + bonding.

*Polycarbonate brackets*: HF + bonding (no silane), phosphoric acid 37% + bonding (no silane), Cojet (a type of silica coating) + silane (no bonding), abrasion + silane (no bonding), abrasion + silane + bonding.

### 2.4. Risk of Bias Assessment

Since there was no tool available to measure the risk of bias of in vitro studies, a questionnaire was devised by VR from various major “risk of bias” assessment tools to include potential sources of bias relevant to in vitro studies. Two authors assessed the studies in this regard.

### 2.5. Effect Measures

In each study, mean SBS values of treatments other than the control (HF + silane) would be diminished by the control in order to calculate their effect sizes. Therefore, the SBS effect measure for each treatment was the difference between the mean SBS of each group subtracted from the mean SBS of the HF + silane group.

ARI data were aggregated in two different forms. Once, they were dichotomized into low and high ARIs and were treated as odds ratios against the gold standard ARI (HF + silane). In this matter, the 4-score ARIs (0 to 3) were dichotomized into two groups of 0 or 1 (as one group of “failure”) and 2 or 3 (as the other group of “success”). The 5-score ARIs were dichotomized into two groups of “success” (previous groups of 1 to 2) and “failure” (previous groups of 3 to 5). Note that the directions of these two ARI systems are the opposite of each other. In the second method, raw data pertaining to ARI scores of similar groups were summed. And the aggregated ARI scores of each group was compared with the aggregated gold standard ARI, using a chi-square test.

### 2.6. Synthesis Method

Studies were grouped according to the methods of surface roughening. The SBS groups were (1) abrasion-no silane-bonding-metal brackets, (2) abrasion- [29, 35] HF-silane-bonding-metal brackets, (3) abrasion-silane-bonding-metal brackets, (4) Cojet- (a method of silica coating) silane-bonding-metal brackets, (5) diamond bur-no silane-bonding-metal bracket, (6) diamond bur-silane-bonding-metal bracket, (7) Er:YAG laser- (1.6 W, 20 Hz) silane-bonding-metal bracket, (8) HF-no silane-bonding-metal bracket, (9) Nd:YAG laser- (1 W) silane-bonding-metal bracket, (10) phosphoric acid 37%-silane-bonding-ceramic bracket, (11) phosphoric acid 37%-silane-bonding-metal bracket.

All the SBS studies except two had reported mean and standard deviations for all their groups. A study had reported median and the interquartile range instead of mean and standard deviation [30]. A formula ((Q3 − Q1)/1.35) [42] was used to convert the range into standard deviation. Also, another formula ((median = Q1 = Q3)/3) [42] was used to convert the median/quartile information into the mean. A study had reported merely mean SBS values without standard deviations [43], which was excluded from meta-analyses pertaining to SBS. All ARI scores had been reported in a way that raw data could be obtained from the presented data.

All the assessed studies were summarized as tables and also as forest plots. Heterogeneity was assessed using various measures including the *I*^2^ statistic. The source of heterogeneity was not statistically assessed, since the studies were all in vitro and usually no study variables except for the main independent and dependent variables existed in each study, and also because many metasamples were small. For sensitivity analysis, forest plots and sample sizes were visually inspected by two statisticians, and almost no cases of extremities were found. Therefore, no statistical sensitivity analyses deemed necessary. Publication bias was assessed using the Egger regression.

### 2.7. Certainty Assessment

Since there was no method of certainty assessment for in vitro studies, we were limited to reporting the certainty based on what we could obtain from other study types.

### 2.8. Statistical Analyses

As detailed above, effect sizes and 95% confidence intervals (CI) were estimated for SBS values and dichotomized ARI values of different surface treatments in comparison with the treatment “HF + silane + bonding.” Heterogeneity was assessed using various measures, including *I*^2^. Meta-analyses were performed using random-effects and fixed-effects models, depending on the heterogeneity of the metasample. Also meta-analyses of SBS were performed comparing different groups other than the gold standard. Publication bias was assessed using an Egger regression and/or funnel plots. Aggregated ARI scores of each treatment were compared with aggregated ARI scores of the treatment “HF + silane + bonding” using a chi-square test. Each aggregated ARI score was compared with an evenly distributed hypothetical target, using a chi-square goodness-of-fit test. For SBS of each group of each study, a 95% CI was computed. SBS values of each group of each study was compared with the SBS values 6 and 10 MPa (as two optimum SBS thresholds for orthodontic brackets [1]) using a one-sample *t*-test. The software in use was STATA (version 17, StataCorp, College Station, TX, USA). The level of significance was set at 0.05.

## 3. Results

**Figure 1:**
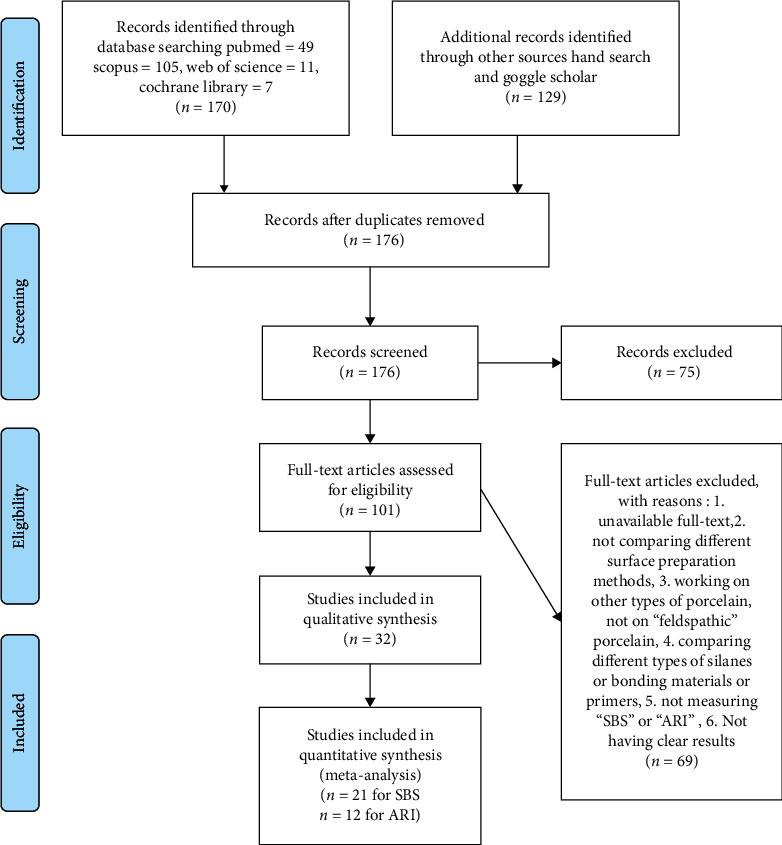
The flow diagram of studies included in this systematic review and meta-analysis.

The search yielded 301 results (49, 105, 11, 7, and 129 in different search engines/databases PubMed, Scopus, Web of Science, Cochrane Library, and Google Scholar, respectively). After finding and removing the duplicates, 176 search results remained. After screening the abstract of these 176 articles, 75 were excluded as not completely relevant. The remaining 101 studies were assessed for the eligibility criteria. Of them, 69 were excluded due to the following reasons: (1) unavailable full text; (2) not comparing different surface preparation methods; (3) working on other types of porcelain, not on “feldspathic” porcelain; (4) comparing different types of silanes or bonding materials or primers; (5) not measuring “SBS” or “ARI”; (6) not having clear results; and (7) comparing different types of silanes or bonding agents which could affect the results of surface treatments as well. There remained 32 studies for qualitative analyses, of which 21 and 12 were included in the quantitative analyses pertaining to SBS and ARI, respectively (Figure 1).

The included studies are summarized (in terms of country, year, sample size, number of groups, brackets, surface roughening methods, silane application protocol, primer application protocol, bonding application protocol, thermocycling, SBS crosshead speed, ARI grouping, and conclusions) in Table 1. All the SBS and ARI values are reported in Tables 2 and 3. Also, statistical comparisons between the gold standard's aggregate ARI and aggregate ARI of each of groups (using the chi-squared test) as well as comparisons between ARI distributions versus an evenly distributed hypothetical target (using the chi-squared goodness-of-fit test) are presented in Table 3.

### 3.1. Summary of Studies That Were Not Included in Meta-Analyses

In the studies that did not have the gold standard group, meta-analysis was not conducted, but their groups were compared with the shear bond strengths 6 and 10 MPa (Table 2). Mirhashemi et al. [44] showed a very high SBS for the silane-less treatment of HF etching and bonding. The three laser groups HF + Er:CrYSGG-3 W-10 Hz + bonding, HF + Er:YAG-3 W-10 Hz + bonding, and Er:CrYSGG-3 W-10 Hz + bonding of their study as well showed very high SBS values. However, another laser group Er:YAG-3 W-10 Hz + bonding yielded merely acceptable (but minimum) results. Cevik et al. [45] did not report adequate information for any statistical calculations. Still, the mean SBS values reported for the gold standard group was mildly greater than phosphoric acid replacement (instead of HF) and mildly poorer than Nd:YAG-15 Hz + silane + bonding treatment, all being extremely low and insufficient. The only group that might provide minimum acceptable shear bond strengths was DB + silane + bonding. Aksakalli et al. [46] showed that the two silane-free treatments HF etching and bonding as well as Er:YAG-2 W-10 Hz laser and bonding could yield quite acceptable results, while another silane-free treatment sandblasting followed by bonding might provide only the minimum required SBS. Again, in the study of Poosti et al. [14], the silane-free group HF etching and bonding was able to provide (this time the minimum) required SBS. Their silane-free laser treatment Nd:YAG-0.8 W + bonding as well provided the minimum necessary SBS. However, the other two laser protocols (2- and 3-watt Er:YAG lasers followed by bonding) failed to do so [14]. Saraç et al. [47] reported high-enough bond strengths for Cojet + silane + bonding and abrasion + silane + bonding, respectively. Karan et al. [48] reported acceptable bond strengths for four treatments “abrasion + silane + bonding, abrasion + HF + bonding, abrasion + HF + silane + bonding, and Cojet + silane + bonding.” However, air abrasion followed by bonding failed to provide proper bond strengths [48]. Tengrungsun et al. [43] had not provided enough information for statistical analyses. Still, all of their three groups (HF + silane + bonding, abrasion + silane + bonding, and Nd:YAG-3 W-20 Hz + silane + bonding) seemed to have proper mean SBS values. Özcan et al. [49] tested 5 treatments “HF + bonding, phosphoric acid 37% + bonding, Cojet + silane, abrasion + silane, and abrasion + silane + bonding” on polycarbonate brackets, all of which provide acceptable bond strengths. They were the only study assessing polycarbonate brackets [49]. There was only one study assessing the treatment “abrasion + HF + bonding” in comparison with the gold standard [29]. Since there were no other such studies, it was not possible to conduct a meta-analysis on it. That study [29] showed that this treatment (abrasion + HF + bonding) was poorer than its control and could provide bond strengths about 1.9 MPa weaker.

### 3.2. Meta-Analyses of SBS

Detailed information of analyses is presented as Figures 2–14. Therefore, we did not repeat most of this information in the text.

#### 3.2.1. SBS of Metal Brackets

**Figure 2:**
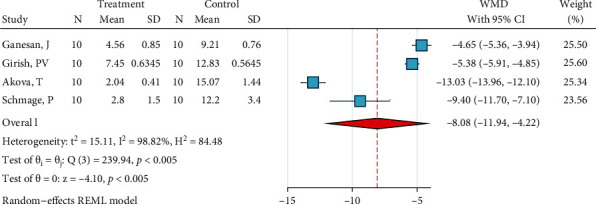
Weighted mean differences (and 95% CIs) for the SBS values produced by the surface treatment “abrasion, no silane, bonding” versus the gold standard for metal brackets.

*(1) Abrasion, No Silane, and Bonding*. Four studies were included in this meta-analysis [2, 29, 31, 34]. The metasample was heterogenous (*I*^2^ = 98.8%, *P* < 0.0005). The overall effect size was significantly below zero, indicating that this treatment is significantly less effective than the gold standard (Figure 2). The Egger regression showed that there was no publication bias across the studies (*P* = 0.554).

**Figure 3:**
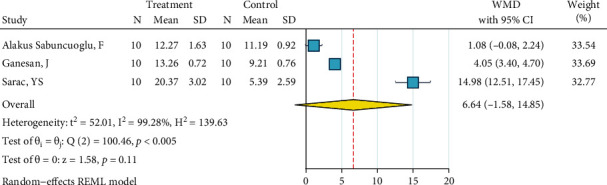
Weighted mean differences (and 95% CIs) for the SBS of “abrasion, HF, silane, bonding” against the gold standard for metal brackets.

*(2) Abrasion, HF, Silane, and Bonding*. Three studies were included in this meta-analysis [27, 29, 35]. The metasample was heterogenous (*I*^2^ = 99.3%, *P* < 0.0005). The overall effect size was marginally significantly above zero, indicating that this treatment might be more effective than the gold standard (Figure 3). The Egger regression showed a marginally significant publication bias (*P* = 0.061).

**Figure 4:**
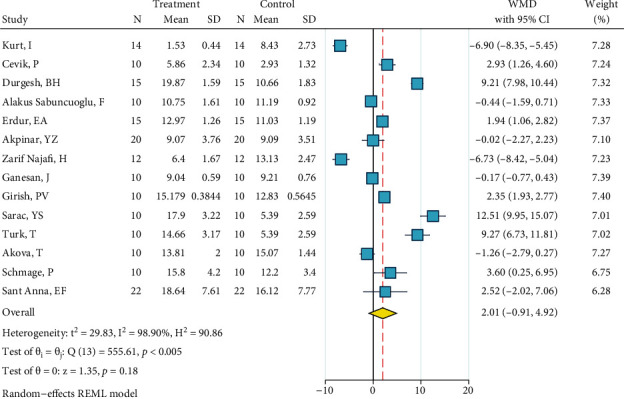
Weighted mean differences (and 95% CIs) for the SBS of “abrasion, silane, bonding” versus the gold standard in metal brackets.

*(3) Abrasion, Silane, and Bonding*. Fourteen studies were included in this meta-analysis [2, 22, 27, 29–39]. The metasample was heterogenous (*I*^2^ = 98.9%, *P* < 0.0005). The overall effect size was almost (marginally significantly) above zero for about 2 MPa, with a very subtle overlap of its 95% CI with the zero line, indicating that taking into account the controversies in the 14 studies, this treatment is, overall, marginally significantly more effective than the gold standard for about 2 MPa (Figure 4). No publication bias was detected (*P* = 0.719).

**Figure 5:**
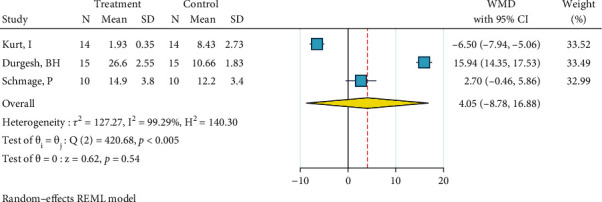
Weighted mean differences (and 95% CIs) of the SBS of “Cojet, silane, bonding” against the gold standard for metal brackets.

*(4) Cojet, Silane, and Bonding*. Three studies were included in this meta-analysis [30, 34, 36]. The metasample was heterogenous (*I*^2^ = 99.5%, *P* < 0.0005). The overall effect size was not different from zero, indicating that this surface treatment might yield results similar to the gold standard, also noting that previous results were controversial (Figure 5). No publication bias was detected (Egger, *P* = 0.979).

**Figure 6:**
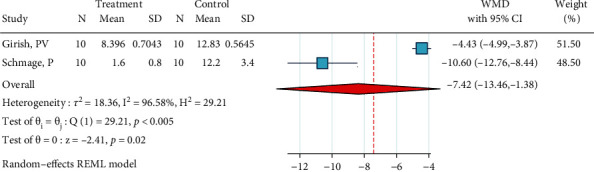
Weighted mean SBS differences (and 95% CIs) of “diamond bur, no silane, bonding” in comparison with the gold standard in metal brackets.

*(5) Diamond Bur, No Silane, and Bonding*. Only two studies were included in this regard [31, 34]. The metasample was heterogenous (*I*^2^ = 96.6%, *P* < 0.0005). The overall effect size was significantly negative, indicating that this surface treatment acts poorer than the gold standard (Figure 6). The Egger regression could not be performed, but the funnel plot indicated a lack of publication bias.

**Figure 7:**
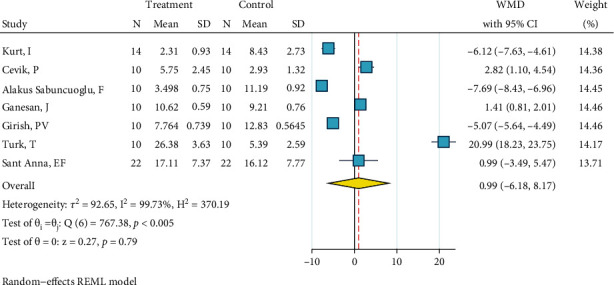
Weighted mean SBS differences (and 95% CIs) of “diamond bur, silane, bonding” compared with the gold standard for metal brackets.

*(6) Diamond Bur, Silane, and Bonding*. Seven studies were included in this meta-analysis [22, 27, 29–33]. The metasample was heterogenous (*I*^2^ = 99.7%, *P* < 0.0005). Most studies were either similar to the control or poorer than it. However, one study had an extremely higher SBS compared with the control [32]. The overall effect size was very close to zero with 95% CIs spanning around zero and thus not significantly different from zero, indicating the possible similarity of this surface treatment with the gold standard as well as some controversy (Figure 7). No publication bias was observed (Egger, *P* = 0.250).

**Figure 8:**
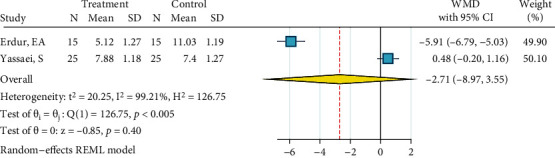
Weighted mean SBS differences (and 95% CIs) for “Er:YAG laser (1.6 W, 20 Hz), silane, bonding” versus the gold standard in metal brackets.

*(7) Er:YAG Laser (1.6 W, 20 Hz), Silane, and Bonding*. Two studies were included [37, 50]. The metasample was heterogenous (*I*^2^ = 99.2%, *P* < 0.0005). The overall effect size was not significantly different from zero, indicating that this treatment might act like the gold standard and/or that the results might be controversial (Figure 8). The Egger regression could not be performed. The funnel plot suggested a lack of publication bias.

**Figure 9:**
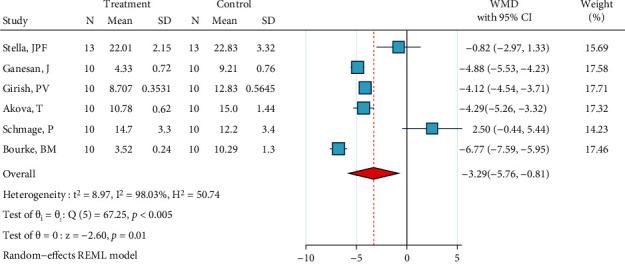
Weighted mean differences (95% CIs) for the SBS of “HF, no silane, bonding” against the gold standard for metal brackets.

*(8) HF, No Silane, and Bonding*. This meta-analysis included six studies [2, 25, 26, 29, 31, 34]. The metasample was heterogenous (*I*^2^ = 98.0%, *P* < 0.0005). The overall effect size was significantly negative, indicating that this surface treatment acted poorer than the gold standard (Figure 9). A significant publication bias was detected (beta = 5.79, SE = 1.610, *P* = 0.0003, Egger regression).

**Figure 10:**
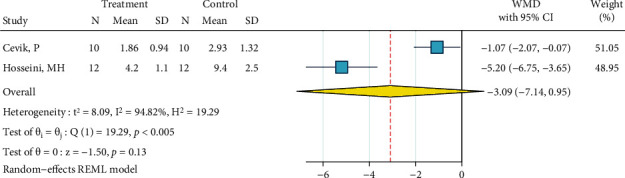
Weighted mean differences (95% CIs) for the bond strengths produced by “Nd:YAG laser (1 W), silane, bonding” versus the gold standard in metal brackets.

*(9) Nd:YAG Laser (1 W), Silane, and Bonding*. This meta-analysis included two studies [12, 22]. The metasample was heterogenous (*I*^2^ = 94.8%, *P* < 0.0005). The overall effect size was about -3.1 MPa, but the very wide 95% CI crossed the zero line with a small margin, rendering the effect size nonsignificant (Figure 10). Still, the overall difference was marginally significant, and this treatment seems to act somehow poorer than the gold standard, as both the effect sizes of both studies and their 95% CIs were all negative (Figure 10). No publication bias was observed (Egger, beta = 0.0).

**Figure 11:**
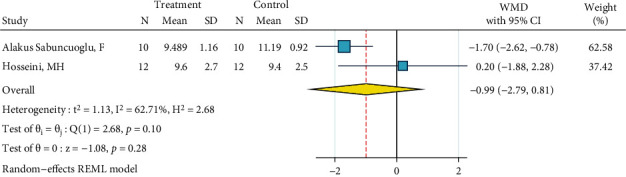
Weighted mean SBS differences (95% CIs) of “Nd:YAG laser (2 W), silane, bonding” protocol in comparison with the gold standard for metal brackets.

*(10) Nd:YAG Laser (2 W), Silane, and Bonding*. This meta-analysis contained two studies [12, 27]. The metasample was not significantly heterogenous (*I*^2^ = 62.7%, *P* = 0.10). The overall effect size was not significantly different from zero (Figure 11). No publication bias was observed (Egger, beta = 0.0).

**Figure 12:**
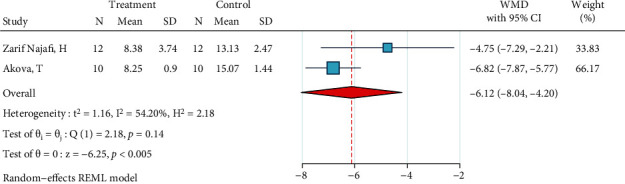
Weighted mean differences (95% CIs) for the SBS values caused by the preparation method “CO_2_ laser (2 W, 2 Hz), silane, bonding” against the gold standard for metal brackets.

*(11) CO_2_ Laser (2 W, 2 Hz), Silane, and Bonding*. This meta-analysis had two studies [2, 39]. The metasample was not significantly heterogenous (*I*^2^ = 54.2%, *P* = 0.14). The significant overall effect size was about -6.1 MPa, indicating the poor quality of this treatment (Figure 12). The Egger test could not be conducted, but the funnel plot did not show any publication bias.

**Figure 13:**
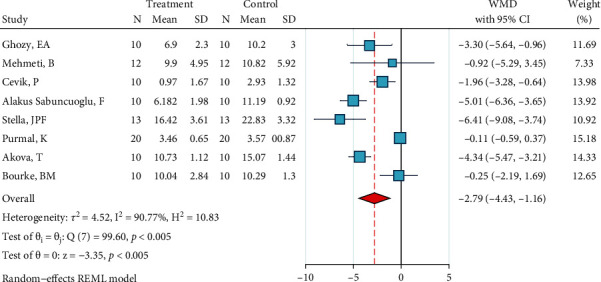
Weighted mean SBS differences (95% CIs) of “phosphoric acid 37%, silane, bonding” versus the gold standard for metal brackets.

*(12) Phosphoric Acid 37%, Silane, and Bonding*. This meta-analysis included eight studies [2, 22–28]. The metasample was heterogenous (*I*^2^ = 90.8%, *P* < 0.0005). The overall effect size of -2.8 MPa was significantly below zero, indicating that this surface treatment acted poorer than the gold standard (Figure 13). There was no publication bias across the studies (*P* = 0.755, Egger).

#### 3.2.2. SBS of Ceramic Brackets

**Figure 14:**
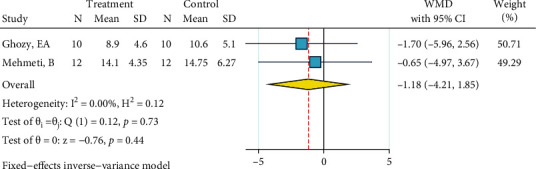
Weighted mean SBS differences (95% CIs) of “phosphoric acid 37%, silane, bonding” compared to the gold standard for ceramic brackets.

*(1) Phosphoric Acid 37%, Silane, and Bonding*. The metasample of the included two studies [23, 24] was homogenous (*I*^2^ = 0.0%, *P* = 0.734). Both studies similarly showed effect sizes close to the gold standard. The overall effect size was not significantly different from zero (Figure 14). The Egger test and funnel plot showed no publication bias (*P* = 0.734).

### 3.3. Meta-Analyses of Dichotomized ARIs

After dichotomizing ARI scores as detailed above, their odds ratios against the dichotomized ARI of the control group (the gold standard) were calculated. Detailed analysis parameters are illustrated as Figures 15–22; therefore, they are not repeated as text.

#### 3.3.1. 4-Score ARIs

*(1) ARI of Metal Brackets*

**Figure 15:**
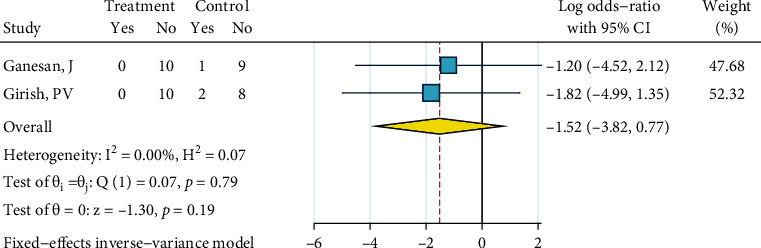
Log odds ratios (and 95% CI) for dichotomized 4-score ARIs for “abrasion, no silane, bonding” versus the gold standard, in metal brackets.

**Figure 16:**
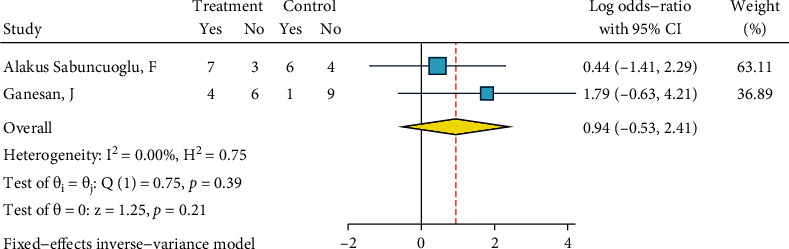
Log odds ratios (95% CI) for dichotomized 4-score ARIs for “abrasion, HF, silane, bonding” against the gold standard for metal brackets.

**Figure 17:**
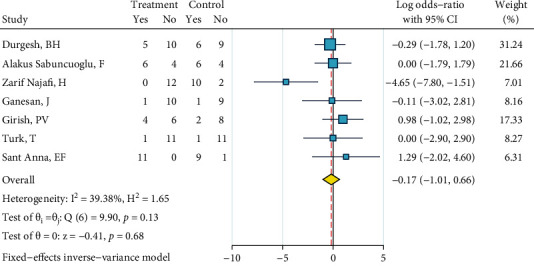
Log odds ratios (95% CI) for dichotomized 4-score ARIs for “abrasion, silane, bonding” compared to the gold standard, in metal brackets.

**Figure 18:**
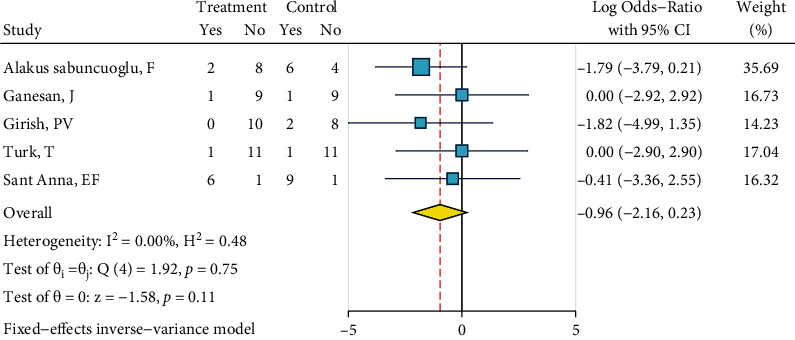
Log odds ratios (95% CI) for dichotomized 4-score ARI for “diamond bur, silane, bonding” against the gold standard, in metal brackets.

**Figure 19:**
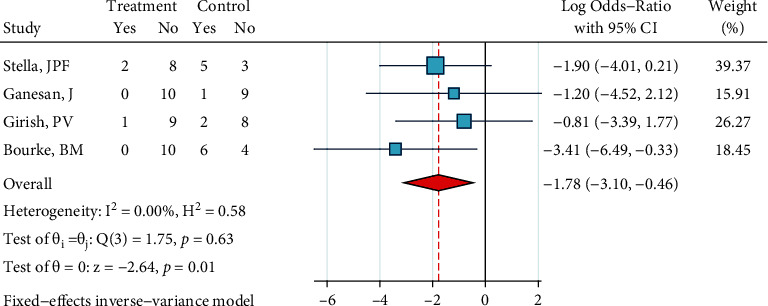
Log odds ratios (95% CI) for dichotomized 4-score ARI for “HF, no silane, bonding” versus the gold standard, in metal brackets.

**Figure 20:**
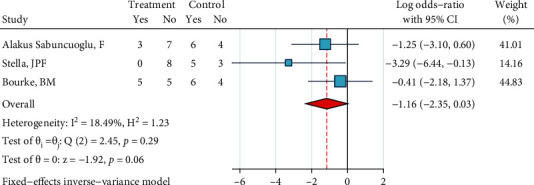
Log odds ratios (95% CI) for dichotomized 4-score ARI for “phosphoric acid 37%, silane, bonding” compared to the gold standard, in metal brackets.

1. *Abrasion, No Silane, and Bonding*. Only two studies were included in this meta-analysis [29, 31]. There was no heterogeneity (*I*^2^ = 0.0%, *P* = 0.791). The overall odds ratio did not differ significantly from the odds ratio = 1, meaning that the dichotomized ARI of these studies did not differ considerably from the dichotomized ARI of the gold standard (Figure 15). The Egger regression and the funnel plot did not show any publication bias (*P* = 0.791).
2. *Abrasion, HF, Silane, and Bonding*. Only two studies were included in this meta-analysis [27, 29]. There was no heterogeneity (*I*^2^ = 0.0%, *P* = 0.386). The overall odds ratio did not differ significantly from the odds ratio = 1, meaning that the dichotomized ARI of these studies did not differ considerably from the dichotomized ARI of the gold standard (Figure 16). The Egger regression and the funnel plot showed no publication bias (*P* = 0.386).
3. *Abrasion, Silane, and Bonding*. Seven studies were included in this meta-analysis [27, 29, 31–33, 36, 39]. There was no heterogeneity (*I*^2^ = 39.4%, *P* = 0.129). The overall odds ratio did not differ significantly from 1 (Figure 17). The Egger regression showed no publication bias (*P* = 0.483).
4. *Diamond Bur, Silane, and Bonding*. This meta-analysis included five studies [27, 29, 31–33]. There was no heterogeneity (*I*^2^ = 0.0%, *P* = 0.751). The overall odds ratio was marginally significantly smaller than 1 (Figure 18), indicating that ARI scores of this treatment might be lower values than the ARI of the gold standard. No publication bias was observed (Egger, *P* = 0.406).
5. *HF, No Silane, and Bonding*. This meta-analysis included four studies [25, 26, 29, 31]. There was no heterogeneity (*I*^2^ = 0.0%, *P* = 0.626). The overall odds ratio was significantly smaller than 1 (Figure 19), indicating that ARI scores of this treatment tend to skew towards the value zero compared to the ARI of the gold standard. There was no publication bias (Egger, *P* = 0.849).
6. *Phosphoric Acid 37%, Silane, and Bonding*. This meta-analysis included three studies [25–27]. There was no significant heterogeneity (*I*^2^ = 0.0%, *P* = 0.293). The overall odds ratio was marginally significantly smaller than 1 with an extremely small margin (*P* = 0.055, Figure 20), indicating that ARI scores of this treatment tend to skew towards the lower end compared to the ARI scores of the gold standard. There was not any case of publication bias (Egger, *P* = 0.141).

#### 3.3.2. 5-Score ARIs

When dichotomizing 5-score ARIs, the higher scores were categorized as failures and the lower scores were categorized as successes. This was opposite of the categories of the 4-score ARIs, as the definitions of the 4- and 5-score ARIs were reverse of each other.

**Figure 21:**
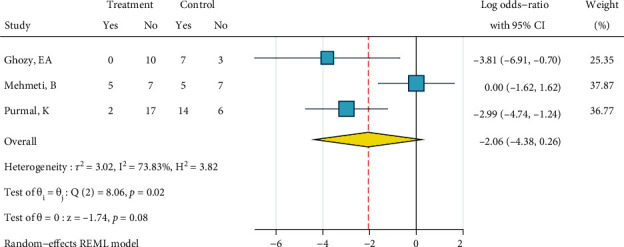
Log odds ratios (95% CI) for dichotomized 5-score ARI for “phosphoric acid 37%, silane, bonding” against the gold standard, in metal brackets. When dichotomizing 5-score ARIs, the higher scores were categorized as failures and the lower scores were categorized as successes (the opposite of 4-score ARIs).

*(1) Metal Brackets: Phosphoric Acid 37%, Silane, and Bonding*. This meta-analysis included three studies [23, 24, 28]. There heterogeneity was significant (*I*^2^ = 73.8%, *P* = 0.018). The overall odds ratio was marginally significantly smaller than 1 (Figure 21). No publication bias was detected (*P* = 0.346).

**Figure 22:**
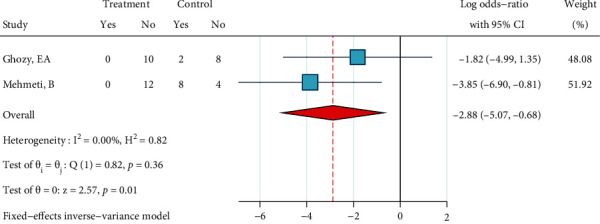
Log odds ratios (95% CI) for dichotomized 5-score ARI for “phosphoric acid 37%, silane, bonding” in comparison with the gold standard for ceramic brackets. When dichotomizing 5-score ARIs, the higher scores were categorized as failures and the lower scores were categorized as successes (the opposite of 4-score ARIs).

*(2) Ceramic Brackets: Phosphoric Acid 37%, Silane, and Bonding*. Only two studies existed that had tested this treatment [23, 24]. No heterogeneity was observed (*I*^2^ = 0.0%, *P* = 0.365). The overall odds ratio was significantly smaller than 1 (Figure 22), indicating that ARI scores of this treatment tend to skew towards the “porcelain-resin junction fracture” end compared to the ARI of the gold standard. The Egger regression and funnel plot did not show any publication bias (*P* = 0.365).

### 3.4. Meta-Analyses of SBS Comparing Groups Other than Gold Standard with Each Other

**Figure 23:**
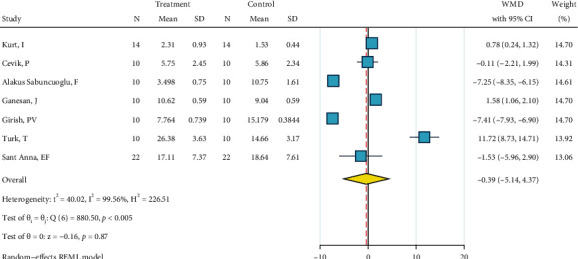
Comparing abrasion, silane, and bonding (as the control) versus diamond bur, silane, and bonding.

*(1) Metal Brackets: Abrasion, Silane, and Bonding (as the Control) versus Diamond Bur, Silane, and Bonding*. A total of 7 studies were included in this meta-analysis [22, 27, 29–33]. The sample was heterogenous. The overall difference between the two methods was close to zero (Figure 23). No publication bias was observed (Egger, *P* = 0.363).

### 3.5. Risk of Bias

**Figure 24:**
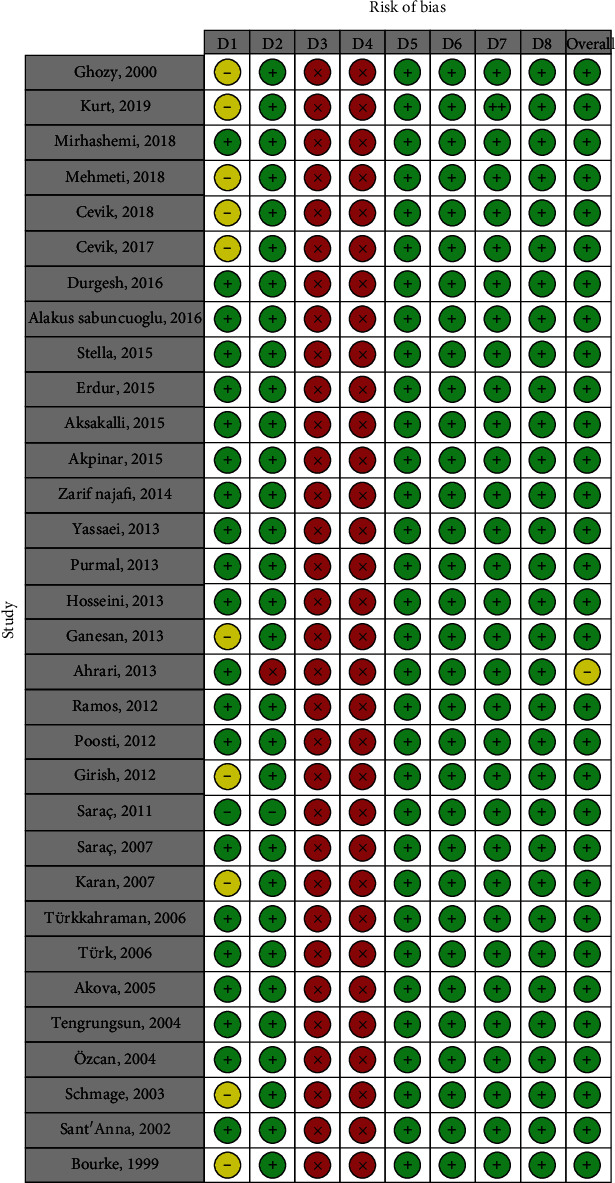
The assessment of the risk of bias. Red crosses: high risks of bias; yellow hyphens: unclear; green pluses: low risks of bias. Domains: (D1) Was there adequate randomization? (D2) Were baseline conditions similar across different groups? (D3) Were experimental procedures similar for different groups? (D4) Were operators blinded to the grouping? (D5) Were outcome data complete without missing? (D6) Were all measured outcomes adequately reported? (D7) Were there any reports of outcomes that were not adequately explained in methods? (D8) Any other inconsistency or source of bias.

**Figure 25:**
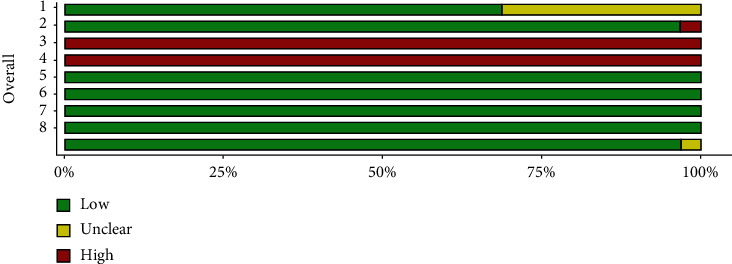
Frequency of risks of biases in each domain. Domains: (D1) Was there adequate randomization? (D2) Were baseline conditions similar across different groups? (D3) Were experimental procedures similar for different groups? (D4) Were operators blinded to the grouping? (D5) Were outcome data complete without missing? (D6) Were all measured outcomes adequately reported? (D7) Were there any reports of outcomes that were not adequately explained in methods? (D8) Any other inconsistency or source of bias.

The risk of bias was assessed using a questionnaire created for in vitro studies. Most studies had adequately randomized the specimens. Baseline conditions were similar across groups for most studies. Experimental procedures were always different across groups. No operators were blinded of the groupings. There was no attrition bias. All the intended outcomes had been adequately reported in all studies. No undefined outcomes had been reported. The summary of these assessments is presented in Figures 24 and 25 and Table 4. Overall, it seemed that most studied had rather low risks of bias.

### 3.6. Certainty of Evidence

There was no method of assessing the certainty of evidence for in vitro studies. In the pyramid of evidences, in vitro studies are at the lowest level because they cannot be translatable to the extremely dynamic oral and systemic conditions. On the other hand, they remove numerous confounding factors in an experimental setting, allowing a better control over results and risks of bias (as was the case with the studies included in this systematic review). Therefore, it seems that their results can be considered moderately confident per se, if not tending to generalize them to clinical conditions.

## 4. Discussion

**Figure 26:**
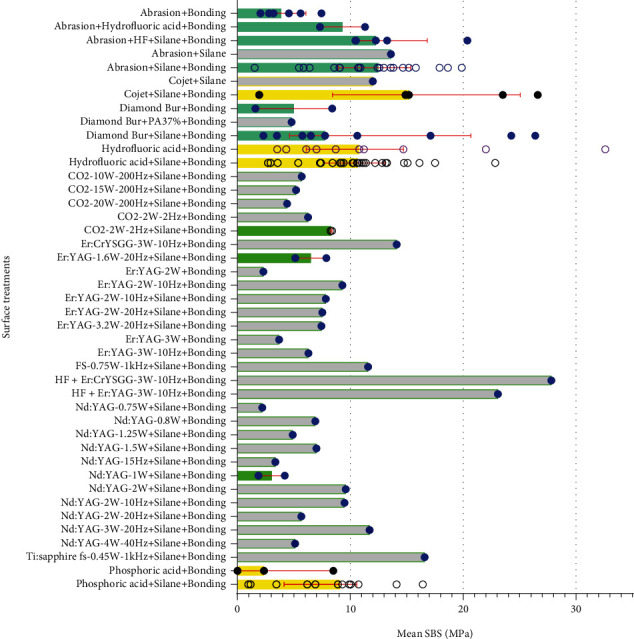
Mean shear bond strengths reported in 140 groups show quite controversial results for many surface treatments. Each circle shows the mean SBS reported by a certain study. Each bar represents the median of the mean SBS values across different studies; this bar is transparent when there is only one study within a given category. Error bars represent interquartile ranges. Color changes are merely for better identification of different parent categories.

The goal of this systematic review and meta-analysis was to offer proper methods that can act as good as the gold standard (HF followed by silane and bonding agent application) or possibly better than it in producing proper shear bond strengths of orthodontic brackets to feldspathic porcelain surfaces. Findings of the above meta-analyses should be approached with caution, as most of them had rather small metasamples and many of the overall effect sizes were not statistically significant. Besides, it should be noted that factors such as the physicochemical properties of materials in use, details of methods such as duration or percentage of HF etching, or even brands of materials (e.g., different silane brands) might affect the outcome [51–53]. Furthermore, it should be noted that although the surface treatment “HF etching followed by silane and bonding application” was used as the gold standard, an inspection of this group itself shows that in some cases, even this gold standard had not reached proper SBS values (Figure 26, Table 2), which can be due to numerous methodological factors such as the number of thermal cycles, the brands and types and concentrations of the materials in use, and durations of application. Another important point to consider is that acting poorer than the gold standard does not always and necessarily mean *failure*. It is possible for a particular surface treatment to act slightly (but significantly) poorer than the gold standard while still providing adequate shear bond strengths. This is why we also compared each group of each study with the values 6 and 10 MPa as the two recommended bond strengths (Table 2) [1]. The literature does not contain clear guidelines about shear force limits [54]. Still, a proper orthodontic biomaterial needs to provide good adhesion in order to endure masticatory forces (with a minimum bond strength of 6 to 10 MPa) [1, 54]. Conversely, adhesion forces should not be excessively strong in order to avoid enamel loss or porcelain damage after debonding (perhaps a maximum of 40 to 50 MPa for certain (and not all) dental tissues but not known for porcelain surfaces) [2, 8, 54, 55]. Therefore, Scribante et al. [54] suggest that an ideal orthodontic material should produce bonding forces between about 5 and 50 MPa, even if these limits are mostly theoretical [54]. It should be noted that even some dental surfaces might not tolerate high bond strengths; for example, enamel tested transversally to its prismatic orientation might bear up to 11.5 MPa only [55]. However, if the tensile force was parallel to the enamel prisms, it could tolerate 42.2 MPa [55]. And it should be also taken into consideration that values suggested as safe for dental tissues might not necessarily apply to porcelains.

Overall, it might be concluded with some degree of confidence that the following surface treatments might provide shear bond strengths significantly lower than the gold standard method: “abrasion + bonding, diamond bur + bonding, HF + bonding, Nd:YAG laser (1 W) + silane + bonding, CO_2_ laser (2 W, 2 Hz) + silane + bonding, and phosphoric acid 37% + silane + bonding.” On the other hand, the addition of sandblasting to the gold standard treatment (abrasion, HF, silane, bonding) might improve the SBS. Moreover, replacing HF etching in the gold standard treatment with sandblasting (abrasion, silane, bonding) would yield quite controversial results (i.e., all the three categories: poorer than gold standard, similar to it, or better than it) that overall might provide marginally significantly better shear bonds than the gold standard for about 2 MPa. Some surface treatments had controversial results with their overall effects being close to the gold standard; these were “Cojet + silane + bonding, diamond bur + silane + bonding, and Er:YAG laser (1.6 W, 20 Hz) + silane + bonding.” And some surface treatments were similar to the gold standard without much controversy: “phosphoric acid 37% + silane + bonding” in ceramic brackets and “Nd:YAG laser (2 W) + silane + bonding” in metal brackets.

The bond should not be too strong and damage porcelain integrity following debonding [8]. Bracket debonding may cause four different fracture types: cohesive within the adhesive layer, cohesive within the porcelain, adhesive at the adhesive–bracket interface, and adhesive at the porcelain–adhesive interface [56]. ARI scores may depend to some degree on the bond strength, but several other factors as well can influence them including adhesive type and bracket base design [57]. It is important to minimize the risk of porcelain damage, and the mode of failure is critical in this matter [58]. Two different ARI definitions had been used by various studies; therefore, we had to investigate each of these two ARI systems separately. Still, we made sure that in both the opposite ARI definitions, the “porcelain-resin junction fracture” was treated as the failure. The assessment of dichotomized ARI scores showed that overall odds ratios for three treatments (produced against the gold standard) were not significant from OR = 1: “abrasion (without silanization) + bonding, abrasion + HF + silane + bonding, and abrasion + silane + bonding.” These were similar to the gold standard. Five of the treatments had dichotomized ARIs towards the porcelain-resin detachment (lower scores in 4-score ARIs and higher scores in 5-score ARIs) compared to the gold standard (diamond bur + silane + bonding, HF (without silane) + bonding, phosphoric acid 37% + silane + bonding in 4-score ARI systems, phosphoric acid 37% + silane + bonding in 5-score ARI systems [which showed a marginally significant overall effect size], and phosphoric acid 37% + silane + bonding for ceramic brackets). No surface treatments tended to have ARI scores skewed towards the “bracket-resin junction fracture” scores (score 4 or 1 in the 4-score or 5-score systems, respectively) compared to the gold standard. Aggregated ARI scores of the gold standard groups were mostly evenly distributed (Table 3). The “HF + bonding” group had ARI scores mostly skewed to the lower end (no resin remnant on the porcelain surface) and different from that of the gold standard in the case of metal brackets. The aggregated ARI scores of the groups “PA37% + silane + bonding,” “abrasion + bonding,” “abrasion + silane + bonding,” “abrasion + HF + bonding,” “Cojet + silane + bonding,” and “Cojet + silane + bonding” were all skewed to the “porcelain-resin adhesive fracture” end (no resin remnant on the porcelain surface), most of which were different from the distribution of the aggregated ARI scores of the gold standard (Table 3).

HF acid may be utilized to chemically alter the porcelain surface [8, 53], and its 9.6% concentration may be advantageous over its 5% concentration [52]. The use of 9.6% HF acid gel has been the most common, but due to its high strength and tissue damage, its use should be controlled [4, 23]. Silanization (after surface preparation and before bonding agent application) as well seems to be one of the factors that can increase the bonding adhesion to the tooth surface or restoration [59]. The findings of this study indicated that without the application of silane, the overall SBS values of different methods (i.e., abrasion + bonding, diamond bur + bonding, and HF + bonding) were all significantly lower than the gold standard. Therefore, it seems that silanization might be an important and necessary step of the process. However, it might not be the only necessary step, as we observed that few other surface treatments including silane as well failed to produce SBS values as strong as the gold standard (Nd:YAG laser (1 W) + silane + bonding or phosphoric acid 37% + silane + bonding). Silane is a coupling agent composed of bifunctional molecules which bond to porcelain on the one end and to resin on the other end, enhancing the bond to bracket, not to mention that it improves the wettability of porcelain surface as well [56, 60]. Earlier studies regarding silane effects on SBS of brackets to porcelains were controversial [47, 52, 56, 60, 61] as some of them have found no advantage for silane application [48, 61].

Instead of HF or besides it, some other methods can be used to etch or roughen the porcelain surface. If useful, these might reduce some problems associated with HF preparation, application, and expiration. Porcelain surface can be roughened via sandblasting or burring [2, 22, 27, 29–39]. Earlier studies on the efficacy of one of these two methods over the other are controversial. Our meta-analysis showed that overall, these two methods (together with silane and bonding application) might have similar efficacies in attaching metal brackets to porcelain and also that both of these may act almost similar to the gold standard; sandblasting might even slightly improve the overall SBS compared to the gold standard. Another method replacing HF etching is Cojet, which was shown to produce overall SBS values close to those of the gold standard despite the controversy observed over the three studies [30, 34, 36]. Some studies have suggested the use of phosphoric acid instead of HF [2, 22–28]; although the two available studies on ceramic brackets showed an overall SBS similar to that of the gold standard [23, 24], most studies on metal brackets showed bond strengths weaker than those of the gold standard [2, 22–28]. The results of those two studies on ceramic brackets should be approached with caution as well, as both of them were slightly poorer than the gold standard [23, 24]. Another HF replacement may be laser irradiation; it can roughen the surface via concentrated heat, depending on the energy of its photons [44]. Overall, it was found that the laser irradiation protocols that might be somehow comparable to the gold standard in some cases were Er:YAG (1.6 W, 20 Hz) and Nd:YAG (2 W) (both followed by silane and bonding agent application) [12, 27, 37, 50]. It should be noted that in each of these two protocols, one of the two available studies was similar to the gold standard (in terms of SBS) while the other one was severely weaker than it [12, 27, 37, 50]. The Nd:YAG laser (1 W) protocol had two studies, both acting poorer than the gold standard [12, 22]. Similarly, CO_2_ laser (2 W, 2 Hz) was tested in two studies, both failing to produce proper bond strengths [2, 39]. So if the goal is to produce bonds as strong as those produced by the gold standard, perhaps the Er:YAG (1.6 W, 20 Hz) and Nd:YAG (2 W) lasers might be used instead of HF, after proper optimizations. Some other laser protocols might provide proper or even high bond strengths. For example, the lasers used by Mirhashemi et al. [44] should be further investigated. Since they [44] had not adopted the gold standard, their results were not meta-analyzed, but the lasers used by them (Er:CrYSGG-3 W-10 Hz and Er:YAG-3 W-10 Hz followed by bonding even without silanization) showed quite appropriate bond strengths without HF application, while resulting in very high bond strengths when combined with HF etching [44]. There were other laser protocols as well which would benefit from further investigation, since they were able to produce proper bonds (CO_2_-2 W-2 Hz + Si + Bo, CO_2_-10 W-200 Hz + Si + Bo, CO_2_-15 W-200 Hz + Si + Bo, CO_2_-2 W-2 Hz + Bo, Er:CrYSGG-3 W-10 Hz + Bo, Er:YAG-1.6 W-20 Hz + Si + Bo, Er:YAG-2 W-10 Hz + Bo, Er:YAG-2 W-10 Hz + Si + Bo, Er:YAG-2 W-20 Hz + Si + Bo, Er:YAG-3.2 W-20 Hz + Si + Bo, Er:YAG-3 W-10 Hz + Bo, FS-0.75 W-1 kHz + Si + Bo, Nd:YAG-0.8 W + Bo, Nd:YAG-1.25 W + Si + Bo, Nd:YAG-1.5 W + Si + Bo, Nd:YAG-2 W + Si + Bo, Nd:YAG-2 W-10 Hz + Si + Bo, Nd:YAG-2 W-20 Hz + Si + Bo, Nd:YAG-3 W-20 Hz + Si + Bo) or even very strong bonds (Ti:sapphire fs-0.45 W-1 kHz + Si + Bo, Er:CrYSGG-3 W-10 Hz + Bo, Table 2).

We also compared each of the groups of each study with the bond strengths 6 MPa and 10 MPa (Table 2, Figure 26). It was found that the following surface treatments might fail to produce adequate bond strengths (that would be insignificantly different from 6 MPa or reasonably greater than it): HF + silane + bonding (the gold standard itself which failed to produce proper bond strengths in 2 studies), Nd:YAG-1 W + silane + bonding (failed in 3 studies), abrasion + bonding (failed in 4 studies), diamond bur + silane + bonding (failed in 2 studies), PA37% + silane + bonding (failed in 3 studies), PA37% + bonding (failed in 2 studies), HF + bonding (failed in 2 studies), Er:YAG-1.6 W-20 Hz + silane + bonding, Nd:YAG-4 W-40 Hz + silane + bonding, Nd:YAG-15 Hz + silane + bonding (mean = 3.37 MPa), Nd:YAG-0.75 W + silane + bonding, CO_2_-20 W-200 Hz + silane + bonding, Er:YAG-2 W + bonding, Er:YAG-3 W + bonding, diamond bur + PA37% + bonding, diamond bur + bonding, abrasion + silane + bonding, Cojet + silane + bonding (each failed in 1 study, Table 2 and Figure 26). On the other hand, the following protocols yielded bond strengths that were higher than 10 MPa: HF + silane + bonding (the gold standard producing excessively high bond strengths in 11 studies), abrasion + silane + bonding (in 11 studies), Cojet + silane + bonding (in 4 studies), abrasion + HF + silane + bonding (in 3 studies), diamond bur + silane + bonding (in 3 studies), HF + bonding (in 3 studies), PA37% + silane + bonding (in 2 studies), HF + Er:CrYSGG-3 W-10 Hz + bonding, HF + Er:YAG-3 W-10 Hz + bonding, Ti:sapphire fs-0.45 W-1 kHz + silane + bonding, Er:CrYSGG-3 W-10 Hz + bonding, and abrasion + silane (each in 1 study, Table 2 and Figure 26). Some of these strong bonds were still rather close to 10 MPa; nevertheless, many of them were very strong (Table 2 and Figure 26). Many surface treatments produced desirable bond strengths between 6 and 10 MPa: HF + silane + bonding (the gold standard, producing appropriate bond strengths in 14 studies), abrasion + silane + bonding (in 9 studies), PA37% + silane + bonding (in 7 studies), HF + bonding (in 6 studies), CO_2_-2 W-2 Hz + silane + bonding (in 2 studies), Diamond bur + silane + bonding (in 4 studies), abrasion + bonding (in 2 studies), abrasion + HF + bonding (in 2 studies), abrasion + HF + silane + bonding (in 2 studies), CO_2_-10 W-200 Hz + silane + bonding, CO_2_-15 W-200 Hz + silane + bonding, CO_2_-2 W-2 Hz + bonding, Cojet + silane, diamond bur + bonding, Er:CrYSGG-3 W-10 Hz + bonding, Er:YAG-1.6 W-20 Hz + silane + bonding, Er:YAG-2 W-10 Hz + bonding, Er:YAG-2 W-10 Hz + silane + bonding, Er:YAG-2 W-20 Hz + silane + bonding, Er:YAG-3.2 W-20 Hz + silane + bonding, Er:YAG-3 W-10 Hz + bonding, FS-0.75 W-1 kHz + silane + bonding, Nd:YAG-0.8 W + bonding, Nd:YAG-1.25 W + silane + bonding, Nd:YAG-1.5 W + silane + bonding, Nd:YAG-2 W + silane + bonding, Nd:YAG-2 W-10 Hz + silane + bonding, Nd:YAG-2 W-20 Hz + silane + bonding, Nd:YAG-3 W-20 Hz + silane + bonding, and PA37% + bonding (each in one study, Table 2 and Figure 26).

As stated above, this review was limited by some factors. Firstly, it overlooked any potential studies published before 1990. Moreover, many of the numerous meta-analyses conducted in this study had small metasamples. Besides, most metasamples were heterogenous, and the results could differ based on numerous methodological variables. Sensitivity analyses were merely done subjectively and by visual inspection of forest plots. And the source of heterogeneity was not assessed since in vitro studies usually control for many confounding variables. Most importantly, this review summarized results of in vitro studies, which cannot be easily generalized to clinical conditions. Hence, its results should be interpreted with caution. Future clinical studies are warranted to assess the efficacy of the methods marked as appropriate in this review under clinical circumstances. Moreover, future research should adopt more accurate and also standardized ARI scores, instead of the different ARI definitions currently in use. As advantages, the number of studies included in the systematic review and then meta-analyses were appropriate. Furthermore, the SBS values, dichotomized ARIs, and aggregated ARIs of various protocols in a several studies were summarized and, when possible, analyzed statistically. We did not rely merely on comparisons with the gold standard, since the gold standard itself might produce too strong or too weak results in some situations; accordingly, we also calculated 95% CIs for mean bond strengths and also compared all surface treatments with the SBS range recommended for bonding orthodontic brackets [1].

## 5. Conclusions

It is possible to produce quite different shear bond strengths using the same surface treatment protocol, depending on various methodological factors. It can be concluded that

1. Based on the SBS and ARI values, the gold standard method seems one of the best surface treatments in terms of top SBS values; nonetheless, in some occasions, the gold standard approach might produce very strong or even too weak bonds.
2. Some surface treatments might provide shear bond strengths poorer than the gold standard and are not recommended unless optimized to produce bond strengths about at least 6 to 10 MPa: “abrasion + bonding, diamond bur + bonding, HF + bonding, Nd:YAG laser (1 W) + silane + bonding, CO_2_ laser (2 W, 2 Hz) + silane + bonding, and phosphoric acid 37% + silane + bonding”.
3. The addition of sandblasting to the gold standard treatment (becoming abrasion, HF, silane, and bonding) might improve the SBS.
4. Replacing HF etching in the gold standard treatment with sandblasting (becoming abrasion, silane, and bonding) would yield quite controversial results that overall might provide marginally significantly better shear bonds than the gold standard for about 2 MPa.
5. Some methods had controversial results with their overall effects being close to the gold standard; these were “Cojet + silane + bonding, diamond bur + silane + bonding, and Er:YAG laser (1.6 W, 20 Hz) + silane + bonding”.
6. Certain approaches provided bond strengths similar to the gold standard without much controversy: “phosphoric acid 37% + silane + bonding” in ceramic brackets and “Nd:YAG laser (2 W) + silane + bonding” in metal brackets.
7. Sandblasting and bur roughening (each followed by silanization and bonding application) might have similar efficacies in terms of SBS.
8. Silanization might be recommended, as all the nonsilanated groups had poorer bond strengths compared to the gold standard. This needs further analyses focused on this variable.
9. Dichotomized ARI of three methods seemed to be similar to the gold standard ARI: “abrasion (without silanization) + bonding, abrasion + HF + silane + bonding, and abrasion + silane + bonding”.
10. Five of the treatments had dichotomized ARIs towards the porcelain-resin detachment (lower scores in 4-score ARIs and higher scores in 5-score ARIs) compared to the gold standard; these were diamond bur + silane + bonding, HF (without silane) + bonding, phosphoric acid 37% + silane + bonding in 4-score ARI systems, phosphoric acid 37% + silane + bonding in 5-score ARI systems, and phosphoric acid 37% + silane + bonding for ceramic brackets.
11. No surface treatments tended to have ARI scores skewed towards the “bracket-resin junction fracture” scores (score 4 or 1 in the 4-score or 5-score systems, respectively) compared to the gold standard.
12. The aggregated ARI scores of the gold standard groups were mostly distributed evenly. The “HF + bonding” group had ARI scores usually skewed to the lower end (no resin remnant on the porcelain surface) and different from that of the gold standard in the case of metal brackets.
13. The aggregated ARI scores of the groups “PA37% + silane + bonding,” “abrasion + bonding,” “abrasion + silane + bonding,” “abrasion + HF + bonding,” “Cojet + silane + bonding,” and “Cojet + silane + bonding” were all skewed to the “porcelain-resin adhesive fracture” end (no resin remnant on the porcelain surface), most of which were different from the distribution of the aggregated ARI scores of the gold standard.

## Figures and Tables

**Table 1:** Summary of the included studies.

1st author	Country, year	*N*	No. of groups	Brackets	Surface roughening methods	Silane application protocol	Primer application protocol	Bonding application protocol	Thermal cycling	SBS crosshead speed	ARI grouping	Conclusion
[23] Ghozy, E. A.^a^	Egypt, 2020	40	4	MB & CB	9.5% HFA, 37% PA	One layer (SILAN, Cerkamed, Stalowa Wola, Poland) applied and dried	Transbond XT primer (3M Unitek, CA, USA) applied and air-thinned	Transbond XT adhesive paste (3M Unitek, CA, USA) applied and light-cured	1000	0.5 mm/min	1-5^x^	Etching with HFA provided a significantly higher SBS compared to PA. CBs had a significantly higher SBS than MBs.
[30] Kurt, I.^a^	Turkey, 2019	56	4	MB	9.6% HFA, 50 *μ*m ab, 30 *μ*m Si, ultrafine DB	One layer (ESPE-Sil, 3M ESPE, Seefeld, Germany) applied and dried	—	Transbond XT adhesive paste (3M Unitek, CA, USA) applied and light-cured	1000	1 mm/min	—	HFA provided a significantly higher SBS compared to other methods.
[44] Mirhashemi, A.	Iran, 2018	60	5	MB	9% HFA, 9% HFA + Er:CrYSGG (3 W, 10 Hz), 9% HFA + Er:YAG (3 W, 10 Hz, 300 MJ), Er:CrYSGG (3 W, 10 Hz), Er:YAG (3 W, 10 Hz, 300 MJ)	—	Transbond XT primer (3M Unitek, Monrovia California, USA) applied	Transbond XT adhesive paste (3M Unitek, Monrovia California, USA) applied and light-cured	5000	1 mm/min	0-3^y^	HFA showed the highest SBS. No significant difference observed between the SBS of HFA group and HFA + laser groups. HFA + Er:CrYSGG group caused severe damage to the porcelain structure. Er:CrYSGG group, unlike the Er:YAG group, achieved adequate SBS.
[24] Mehmeti, B.	Croatia, 2018	48	4	MB & CB	5% HFA, 37% PA	Silane (Prosil, Dentscare, Joinville, Brazil) was applied	Transbond XT primer (3M Unitek, Monrovia, CA, USA) applied	Transbond XT adhesive paste (3M Unitek, Monrovia, CA, USA) applied and light-cured	5800	1 mm/min	1-5^x^	Only the type of bracket had a significant effect on SBS (CBs > MBs).
[45] Cevik, P.^a^	Turkey, 2018	50	5	CB	37% PA, 9.6% HFA, DB, Nd:YAG (1 W, 15 Hz), 50 *μ*m Ab	Silane (Ormco's Porcelain Primer; Ormco) applied and dried	—	Composite resin cement (Blugloo; Ormco) applied and light-cured	2500	0.5 mm/min	0-3^y^	Ab provided a significantly higher SBS than HFA. Ab and DB provided higher SBS than other groups.
[22] Cevik, P.^a^	Turkey, 2017	50	5	MB	9.6% HFA, 37% PA, Nd:YAG (1 W), 50 *μ*m Ab, DB	Silane (Ormco's Porcelain Primer; Ormco) applied and dried	—	Composite resin cement (Blugloo; Ormco Corp, Glendora, California) applied and light-cured	2500	0.5 mm/min	—	Ab can be recommended for a durable SBS. Thermocycling might have a negative effect on SBS.
[36] Durgesh, B. H.	Saudi Arabia, 2016	45	3	MB	9.6% HFA, 50 *μ*m Ab, 30 *μ*m Si	One layer (Sil, 3M ESPE™, Seefeld, Germany) applied and dried	Transbond™ XT primer (3M Unitek, Monrovia, CA, USA) applied and air-thinned	Transbond™ XT resin composite applied and light-cured	20000	1 mm/min	0-3^y^	SBS is influenced by the surface roughness.
[27] Alakus Sabuncuoglu, F.	Turkey, 2016	70	7	MB	DB, 37% PA, 9.6% HFA, 50 *μ*m Ab, 50 *μ*m Ab + HFA, Nd:YAG (2 W, 10 Hz), Er:YAG (2 W, 10 Hz)	Silane (Ortho Solo Sealant, Ormco, Orange, CA, USA) applied and air-thinned	—	Adhesive resin (Enlight Light Cure Adhesive, Ormco, Orange, CA, USA) applied	500	0.5 mm/min	0-3^y^	DB alone did not provide adequate SBS. Ab + HFA provided significantly higher SBS than HF or Ab alone. Both lasers showed higher SBS than HFA and Ab alone.
[26] Stella, J. P. F.^b^	Brazil, 2015	52	4	MB	37% PA, 37% liquid PA, 10% HFA	Silane (Dentsply, Petrópolis, RJ, Brazil) applied	—	Transbond XT adhesive paste (3M Unitek, Monrovia, CA, USA) applied and light-cured	—	0.5 mm/sec	0-3^y^	Highest SBS was obtained via HFA (with or without silane)
[37] Erdur, E. A.^a^	Turkey, 2015	75	5	MB	50 *μ*m Ab, 5% HFA, Nd:YAG (2 W, 20 Hz), Er:YAG (1.6 W, 20 Hz), Ti:sapphire (0.45 W, 1 kHz)	Silane (Monobond-S, Ivoclar, Schaan, Liechtenstein) applied and dried	—	Transbond XT adhesive paste (3M Unitek, Monrovia, CA, USA) applied and light-cured	5000	0.5 mm/min	—	Ti:sapphire provided sufficient SBS and could be used as an alternative to conventional surface preparation techniques, but it also resulted in adhesive failure.
[46] Aksakalli, S.	Turkey, 2015	39	3	MB	50 *μ*m Ab, Er:YAG (2 W, 10 Hz), 9.6% HFA	—	—	A no-mix composite (Unite, 3M Unitek, CA, USA) applied	1000	0.5 mm/min	0-3^y^	Er:YAG and HFA showed the highest acceptable SBS, but Ab did not.
[38] Akpinar, Y. Z.	Turkey, 2015	80	4	MB	50 *μ*m Ab, 9.6% HFA, Nd:YAG (4 W, 40 Hz), FS (0.75 W, 1 kHz)	Silane-coating agent (Monobond S, Ivoclar Vivadent, Schaan, Liechtenstein) applied	—	Opal bond MV adhesive (Opal Orthodontic, South Jordan, UT) applied and light-cured	500	1 mm/min	—	FS laser produced high SBS and it appears to be an effective method.
[39] Zarif Najafi, H.^c^	Iran, 2014	48	4	MB	9.6% HFA, CO_2_ (2 W, 2 Hz), 50 *μ*m Ab	Silane coupling agent (Silane Bond Enhancer; Pulpdent Corp.) applied	—	Adhesive (Transbond XT, 3M Unitek, Monrovia, CA) applied and light-cured	—	1 mm/sec	0-3^y^	HFA provided higher SBS. CO_2_ provided adequate SBS. Regarding the time-consuming process, soft tissue injuries, and the excessive SBS of HFA, CO_2_ is recommended as an alternative.
[50] Yassaei, S.	Iran, 2013	100	4	MB	9.6% HFA, Er:YAG (1.6 W, 20 Hz), Er:YAG (2 W, 20 Hz), Er:YAG (3.2 W, 20 Hz)	Silane (Silane Bond Enhancer, Pulpdent) applied and dried	—	A layer of unfilled resin (Resilience, Ortho Technology) applied and light-cured	500	1 mm/min	—	Er:YAG can be an appropriate alternative to HFA. The lowest power of laser provided the least surface destruction and the highest SBS among different powers of laser. However there is no linear relation between the power of laser and SBS.
[28] Purmal, K.	Malaysia, 2013	40	2	MB	9.6% HFA, 37% PA	2 layers of silane coupling agent (Ormco, Glendora, CA) applied and dried	a thin layer of bonding agent (Transbond XT primer, 3M Unitek, Monrovia, CA) applied	Resin (Transbond XT-3M Unitek, Monrovia, CA) applied and light-cured	1000	1 mm/min	1-5^x^	No significant difference observed in SBS of the two acids. PA would be safer method, and easier to clean after debonding.
[12] Hosseini, M. H.	Iran, 2013	72	6	MB	9.6% HFA, Nd:YAG (0.75 W, 10 Hz), Nd:YAG (1 W, 10 Hz), Nd:YAG (1.25 W, 10 Hz), Nd:YAG (1.5 W, 10 Hz), Nd:YAG (2 W, 10 Hz)	Organosilane (Silane, Bond Enhancer Corp.) applied	Adhesive primer (3M Unitek, California, USA) applied and light-cured	Transbond XT adhesive paste (3M Unitek, CA, USA) applied and light-cured	500	0.5 mm/min	1-5^x^	1.5 W and 2 W powers of Nd:YAG can be used as an alternative to HFA.
[29] Ganesan, J.^b^	India, 2013	80	8	MB	5% HFA, Ab, Ab + 5% HFA, DB	Monobond-S (Ivoclar, Vivadent AG, Bendererstrasse 2, FL-9494 Schann Principality of Liechtenstein) applied	—	Adhesive paste (3M Transbond XT, USA) applied and light-cured	5000	2 mm/min	0-3^y^	Surface preparation without silanization leads to low SBS. DB + silane and Ab + silane provided favorable SBS.
[62] Ahrari, F.^c^	Iran, 2013	80	8	MB	CO_2_ (10 W, 200 Hz), CO_2_ (15 W, 200 Hz), CO_2_ (20 W, 200 Hz), 9.6% HFA	Silane (Silane Bond Enhancer; Pulpdent Corp.) applied	—	Transbond XT adhesive (3M Unitek, Monrovia, California, USA) applied and light-cured	—	1 mm/min	—	HFA produced adequate SBS. Due to significantly higher SBS, CO_2_ is recommended as an alternative to HFA.
[63] Ramos, T.^a,b^	Brazil, 2012	30	3	CB	10% HFA, DB + 37% PA	2 layers of silane (Ceramic Bond Bifix DC, Voco, Germany) applied	—	Composite resin Transbond XT (3M Dental Division, Sumaré, SP, Brazil) applied and light-cured	—	0.5 mm/min	0-3^y^	DB + PA did not provide enough SBS. HFA increased the SBS. HFA + silane produced the highest SBS.
[14] Poosti, M.^a^	Iran, 2012	80	4	MB	9.6% HFA, Nd:YAG (0.8 W), Er:YAG (2 W), Er:YAG (3 W)	—	—	No-mix composite (Unite, 3M Unitek, USA) applied	500	0.5 mm/min	—	Both 2 W and 3 W Er:YAG showed significantly lower SBS than Nd:YAG and HFA.
[31] Girish, P. V.^a,b^	India, 2012	60	6	MB	Fine DB, HFA, 50 *μ*m ab	A thin layer of silane (Ultradent) applied and dried	Transbond XT primer (3M Unitek, Monrovia, California) applied	Transbond XT adhesive (3M Unitek, Monrovia California) applied and light-cured	—	1 mm/min	0-3^y^	Ab + silane produced the highest SBS. HFA, Ab, HFA + silane, and Ab + silane can produce clinically acceptable SBS.
[47] Saraç, Y. S.^a^	Turkey, 2011	40	2	MB	25 *μ*m Ab, 30 *μ*m Si	Silane (Transbond XT; 3M Unitek, Monrovia, California, USA) applied	Adhesive primer (Transbond XT; 3M Unitek, Monrovia, California, USA) applied	Adhesive paste (Transbond XT; 3M Unitek) applied and light-cured	1000	1 mm/min	0-3^y^	Silica coating significantly increases SBS.
[35] Saraç, Y. Ş.^a^	Turkey, 2007	30	3	MB	25 *μ*m Ab, 9.6% HFA, 25 *μ*m Ab +9.6% HFA	Silane (Bond Enhancer; Pulpdent) applied	Adhesive primer (Transbond XT; 3M Unitek, Monrovia, California) applied	Adhesive resin (Transbond XT; 3M Unitek) applied and light-cured	500	1 mm/min	—	All groups had SBS values above the optimal range (6-8 MPa), except HFA. Ab + HFA did not show an advantage over HFA for SBS.
[48] Karan, S.^a,b^	Turkey, 2007	70	5	MB	50 *μ*m Ab, 50 *μ*m Ab + 9.6% HFA, 30 *μ*m Si	Silane (ESPE-Sil, 3M ESPE, Seefeld, Germany) applied and dried	Adhesive primer (Transbond XT, 3M Unitek, Monrovia, California) applied	Transbond XT (3 M Unitek, Monrovia, California) applied and light-cured	500	1 mm/min	0-3^y^	All groups except ab showed sufficient SBS. Si and ab + silane showed the highest SBS, but also the highest cohesive ceramic fracture (adhesive resin mainly remained on surface after debonding).
[64] Türkkahraman, H.	Turkey, 2006	30	3	CB	9.6% HFA, Ab + 9.6% HFA, Ab	Silane (Ormco Porcelain Primer, Glendora, California, USA) applied	—	Composite resin (Light Bond, Reliance Orthodontic Products Inc., Itasca, Illinois, USA) applied and light-cured	500	0.5 mm/min	—	HFA + silane had the highest SBS. Ab + silane provided poor SBS. Ab + HFA + silane did not significantly increase the SBS.
[32] Türk, T.^a^	Turkey, 2006	40	4	MB	25 *μ*m Ab, 50 *μ*m Ab, 9.6% HFA, extra-fine DB (40 *μ*m), fine DB (63 *μ*m)	Silane (Bond Enhancer; Pulpdent, Watertown, Massachusetts, USA) applied	Adhesive primer (Transbond™ XT; 3M Unitek, Monrovia, California, USA) applied	Adhesive paste (Transbond™ XT; 3M Unitek, Monrovia, California, USA) applied and light-cured	500	1 mm/min	0-3^y^	All groups had SBS values above the optimal range (6-8 MPa), except HFA + silane. 25 *μ*m ab resulted in minimal surface damage.
[2] Akova, T.^a,b^	Turkey, 2005	80	8	MB	37% PA, 50 *μ*m Ab, 9.6% HFA, CO_2_ (2 W, 2 Hz)	Silane (Heraus Kulzer, Hanau, Germany) applied	—	A self-curing no-mix adhesive (Rely-a-Bond, Ortho Arch, Schaumburg, IL)	500	1 mm/min	—	2 W CO_2_ provided adequate SBS. Silanization improved SBS.
[43] Tengrungsun, T.^a^	Thailand, 2004	48	3	MB	50 *μ*m Ab, Nd:YAG (3 W, 20 Hz), 9.5% HFA	2 layers of Ormco Porcelain Primer (Ormco, Glendora, CA, USA) applied	—	Adhesive (Ormco) applied	—	0.5 mm/min	—	Different methods can produce micromechanical retention and increase SBS.
[49] Özcan, M.^b^	Finland, 2004	30	5	PB	37% PA, 9.5% HFA, 30 *μ*m Ab, 30 *μ*m Si	Silane (ESPE-Sil, 3M ESPE, Seefeld, Germany) applied	Primer (Ormco, Glendora, California) applied	Transbond XT (3M Monrovia, California) applied and light-cured	1000	1 mm/min	0-3^y^	Silanization in Ab and Si groups, eliminated the need for acid etching, primer, and bonding agent application. HF is still the appropriate method.
[34] Schmage, P.^a,b^	Germany & Netherlands, 2003	60	6	MB	Fine DB, 50 *μ*m Ab, 5% HFA, 30 *μ*m Si	Silane (ESPE-Sil, ESPE, Seefeld, Germany) applied and dried	—	Self-curing composite resin (concise, 3 M, St. Paul, Minnesota) applied	5000	1 mm/min	—	SBS increased significantly by silanization in Ab group, but not in HFA group. Si resulted in the most favorable SBS. Si + silane might be an alternative to other methods.
[33] Sant'Anna, E. F.^a^	Brazil, 2002	66	3	MB	DB, 10% HFA, 50 *μ*m Ab	3 layers of silane (Scotchprime Ceramic Primer, 3M Unitek, Monrovia, CA, USA) applied and dried	—	Concise system	500	1 mm/min	0-3^y^	Although the Ab resulted in highest SBS, this study concluded that with appropriate material selection, the silane/composite procedure alone may be adequate for bonding.
[25] Bourke, B. M.^a,b,c^	UK, 1999	80	8	MB	37% PA, 9.6% HFA	3 coats of Scotchprime silane applied	—	Scotchbond adhesive applied and light-cured	500	5 mm/min	0-3^y^	PA had favorable SBS. Use of silane resulted in satisfactory SBS. The amount of composite resin remaining on the porcelain was independent from the bonding regime.

^a^These studies had other groups which were not relevant to the main question of this study and did not enter the meta-analysis. ^b^In these studies, silane and/or primer was not applied in all groups. ^c^In these studies, groups of glazed porcelain surfaces were included and deglazed porcelain surfaces were excluded. ^∗^In this study, microshear bond strength was assessed. HFA: hydrofluoric acid; PA: phosphoric acid; Ab: abrasion (sandblasting or air abrasion with Al_2_O_3_,); Si: silica coating; DB: diamond bur; Er:CrYSGG: type of laser; Er:YAG: type of laser; Nd:YAG: type of laser; Ti:sapphire: type of laser; FS: femtosecond laser; CO_2_: CO_2_ laser; CB: ceramic bracket; MB: metal bracket; PB: polycarbonate bracket. ^x^1: 100%, 2: >90%, 3: 90-10%, 4: <10%, 5: 0% of the adhesive left on the porcelain surface. ^y^0: 0%, 1: <50%, 2: >50%, 3: 100% of the adhesive left on the porcelain surface.

**Table 2:** Summary of study variables and their SBS and ARI scores. The 95% CIs are calculated for each study group. The *P* values are calculated using the one-sample *t*-test by comparing each group with the SBS = 6 and 10 MPa, as bond strengths recommended for bonding orthodontic brackets.

Study	Surface treatment	Br	*N*	SBS (MPa)						R	ARI^∗^					
				Mean	SD	95% CI		*P*_6_	*P*_10_		Type	A	B	C	D	E
Ghozy, 2020	HF + Si + Bo	MB	10	10.2	3.0	8.1	12.3	0.0017	0.8377	M	5-1	2	0	1	3	4
	PA37% + Si + Bo	MB	10	6.9	2.3	5.3	8.5	0.2472	0.0021	M	5-1	5	5	0	0	0
	HF + Si + Bo	CB	10	10.6	5.1	7.0	14.2	0.0190	0.7185	M	5-1	3	5	0	0	2
	PA37% + Si + Bo	CB	10	8.9	4.6	5.6	12.2	0.0773	0.4689	M	5-1	3	7	0	0	0
Kurt, 2019	HF + Si + Bo	MB	14	8.43	2.73	6.9	10.0	0.0054	0.0508	M						
	Ab + Si + Bo	MB	14	1.53	0.44	1.3	1.8	<0.00005	<0.00005	F						
	Cojet + Si + Bo	MB	14	1.93	0.35	1.7	2.1	<0.00005	<0.00005	F						
	DB + Si + Bo	MB	14	2.31	0.93	1.8	2.8	<0.00005	<0.00005	F						
Mirhashemi, 2018	HF + Bo	MB	12	32.58	9.21	26.7	38.4	<0.00005	<0.00005	H	0-3	1	4	3	3	
	HF+Er:CrYSGG-3 W-10 Hz + Bo	MB	12	27.81	7.66	22.9	32.7	<0.00005	<0.00005	H	0-3	2	7	1	1	
	HF+Er:YAG-3 W-10 Hz + Bo	MB	12	23.08	9.55	17.0	29.1	<0.00005	0.0006	H	0-3	7	3	1	0	
	Er:CrYSGG-3 W-10 Hz + Bo	MB	12	14.11	9.35	8.2	20.1	0.0120	0.1560	M	0-3	10	1	0	0	
	Er:YAG-3 W-10 Hz + Bo	MB	12	6.3	3.09	4.3	8.3	0.7430	0.0016	M	0-3	10	0	1	0	
Mehmeti, 2018	HF + Si + Bo	MB	12	10.82	5.92	7.1	14.6	0.0167	0.6408	M	5-1	1	3	3	2	3
	PA37%+Si + Bo	MB	12	9.9	4.95	6.8	13.0	0.0196	0.9455	M	5-1	1	4	2	3	2
	HF + Si + Bo	CB	12	14.75	6.27	10.8	18.7	0.0005	0.0236	H	5-1	2	2	6	2	0
	PA37% + Si + Bo	CB	12	14.1	4.35	11.3	16.9	<0.00005	0.0075	H	5-1	0	4	8	0	0
Cevik, 2018	HF + Si + Bo	CB	10	2.71						F	0-3	5	1	0	0	
	PA37% + Si + Bo	CB	10	1.17						F	0-3	6	0	0	0	
	Ab + Si + Bo	CB	10	8.58						M	0-3	4	6	0	0	
	DB + Si + Bo	CB	10	6.51						M	0-3	5	5	0	0	
	Nd:YAG-15 Hz + Si + Bo	CB	10	3.37						F	0-3	2	6	0	0	
Cevik, 2017	HF + Si + Bo	MB	10	2.93	1.32	2.0	3.9	<0.00005	<0.00005	F						
	PA37% + Si + Bo	MB	10	0.97	1.67	-0.2	2.2	<0.00005	<0.00005	F						
	Ab + Si + Bo	MB	10	5.86	2.34	4.2	7.5	0.8541	0.0003	M						
	DB + Si + Bo	MB	10	5.75	2.45	4.0	7.5	0.7543	0.0004	M						
	Nd:YAG-1 W + Si + Bo	MB	10	1.86	0.94	1.2	2.5	<0.00005	<0.00005	F						
Durgesh, 2016	HF + Si + Bo	MB	15	10.66	1.83	9.6	11.7	<0.00005	0.1842	M	0-3	3	6	2	4	
	Ab + Si + Bo (25-micron)	MB	15	19.87	1.59	19.0	20.8	<0.00005	<0.00005	H	0-3	5	5	3	2	
	Cojet + Si + Bo	MB	15	26.6	2.55	25.2	28.0	<0.00005	<0.00005	H	0-3	6	7	1	1	
Alakus Sabuncuoglu, 2016	HF + Si + Bo	MB	10	11.19	0.92	10.5	11.8	<0.00005	0.0027	H	0-3	0	4	3	3	
	PA37% + Si + Bo	MB	10	6.182	1.98	4.8	7.6	0.7779	0.0002	M	0-3	1	6	3	0	
	Ab + Si + Bo	MB	10	10.75	1.61	9.6	11.9	<0.00005	0.1748	M	0-3	0	4	4	2	
	Ab + HF + Si + Bo	MB	10	12.27	1.63	11.1	13.4	<0.00005	0.0017	H	0-3	0	3	3	4	
	DB + Si + Bo	MB	10	3.498	0.75	3.0	4.0	<0.00005	<0.00005	F	0-3	1	7	2	0	
	Er:YAG-2 W-10 Hz + Si + Bo	MB	10	7.829	1.49	6.8	8.9	0.0037	0.0013	M	0-3	0	3	5	2	
	Nd:YAG-2 W-10 Hz + Si + Bo	MB	10	9.489	1.16	8.7	10.3	<0.00005	0.1971	M	0-3	0	2	6	2	
Stella, 2015	HF + Si + Bo	MB	13	22.83	3.32	20.8	24.8	<0.00005	<0.00005	H	0-3	2	1	0	5	
	HF + Bo	MB	13	22.01	2.15	20.7	23.3	<0.00005	<0.00005	H	0-3	6	2	0	2	
	PA37% + Si + Bo (gel PA)	MB	13	16.42	3.61	14.2	18.6	<0.00005	<0.00005	H	0-3	5	3	0	0	
	PA37% + Si + Bo (liquid PA)	MB	13	9.29	1.95	8.1	10.5	<0.00005	0.2138	M	0-3	9	2	0	0	
Erdur, 2015	HF + Si + Bo	MB	15	11.03	1.19	10.4	11.7	<0.00005	0.0047	H						
	Ab + Si + Bo	MB	15	12.97	1.26	12.3	13.7	<0.00005	<0.00005	H						
	Er:YAG-1.6 W-20 Hz + Si + Bo	MB	15	5.12	1.27	4.4	5.8	0.0178	<0.00005	F						
	Nd:YAG-2 W-20 Hz + Si + Bo	MB	15	5.67	1.03	5.1	6.2	0.2350	<0.00005	M						
	Ti:sapphire fs-0.45 W-1 kHz + Si + Bo	MB	15	16.59	1.4	15.8	17.4	<0.00005	<0.00005	H						
Aksakalli, 2015	HF + Bo	MB	13	10.8	3.8	8.5	13.1	0.0007	0.4625	M	0-3	0	4	5	4	
	Ab + Bo	MB	13	5.6	2.9	3.8	7.4	0.6280	0.0001	M	0-3	0	5	5	3	
	Er:YAG-2 W-10 Hz + Bo	MB	13	9.3	1.5	8.4	10.2	<0.00005	0.1183	M	0-3	0	3	6	4	
Akpinar, 2015	HF + Si + Bo	MB	20	9.09	3.51	7.4	10.7	0.0009	0.2606	M						
	Ab + Si + Bo	MB	20	9.07	3.76	7.3	10.8	0.0017	0.2825	M						
	Nd:YAG-4 W-40 Hz + Si + Bo	MB	20	5.11	1.53	4.4	5.8	0.0175	<0.00005	F						
	FS-0.75 W-1 kHz + Si + Bo	MB	20	11.58	4.16	9.6	13.5	<0.00005	0.1057	M						
Zarif Najafi, 2014	HF + Si + Bo	MB	12	13.13	2.47	11.6	14.7	<0.00005	0.0011	H	0-3	0	2	10	0	
	Ab + Si + Bo	MB	12	6.4	1.67	5.3	7.5	0.4243	<0.00005	M	0-3	8	4	0	0	
	CO_2_-2 W-2 Hz + Si + Bo	MB	12	8.38	3.74	6.0	10.8	0.0497	0.1616	M	0-3	7	4	1	0	
Yassaei, 2013	HF + Si + Bo	MB	25	7.4	1.27	6.9	7.9	<0.00005	<0.00005	M						
	Er:YAG-1.6 W-20 Hz + Si + Bo	MB	25	7.88	1.18	7.4	8.4	<0.00005	<0.00005	M						
	Er:YAG-2 W-20 Hz + Si + Bo	MB	25	7.52	1.09	7.1	8.0	<0.00005	<0.00005	M						
	Er:YAG-3.2 W-20 Hz + Si + Bo	MB	25	7.45	1.53	6.8	8.1	<0.00005	<0.00005	M						
Purmal, 2013	HF + Si + Bo	MB	20	3.57	0.87	3.2	4.0	<0.00005	<0.00005	F	5-1	2	2	2	8	6
	PA37% + Si + Bo	MB	20	3.46	0.65	3.2	3.8	<0.00005	<0.00005	F	5-1	11	5	2	1	1
Hosseini, 2013	HF + Si + Bo	MB	12	9.4	2.5	7.8	11.0	0.0006	0.4234	M	5-1	2	4	2	3	1
	Nd:YAG-0.75 W + Si + Bo	MB	12	2.2	0.9	1.6	2.8	<0.00005	<0.00005	F	5-1	3	2	4	2	1
	Nd:YAG-1 W + Si + Bo	MB	12	4.2	1.1	3.5	4.9	0.0001	<0.00005	F	5-1	2	2	4	2	2
	Nd:YAG-1.25 W + Si + Bo	MB	12	4.9	2.4	3.4	6.4	0.1407	<0.00005	M	5-1	0	1	4	5	2
	Nd:YAG-1.5 W + Si + Bo	MB	12	7	1.7	5.9	8.1	0.0664	<0.00005	M	5-1	2	5	3	1	1
	Nd:YAG-2 W + Si + Bo	MB	12	9.6	2.7	7.9	11.3	0.0007	0.6180	M	5-1	1	1	5	3	2
Ganesan, 2013	HF + Si + Bo	MB	10	9.21	0.76	8.7	9.8	<0.00005	0.0094	M	0-3	4	5	1	0	
	HF + Bo	MB	10	4.33	0.72	3.8	4.8	<0.00005	<0.00005	F	0-3	10	0	0	0	
	Ab + Bo	MB	10	4.56	0.85	4.0	5.2	0.0005	<0.00005	F	0-3	10	0	0	0	
	Ab + Si + Bo (50-micron ab)	MB	10	9.04	0.59	8.6	9.5	<0.00005	0.0006	M	0-3	4	6	1	0	
	Ab + Si + Bo (sandblast)	MB	10	12.57	0.84	12.0	13.2	<0.00005	<0.00005	H	0-3	5	3	1	1	
	Ab + HF + Bo	MB	10	7.31	0.83	6.7	7.9	0.0007	<0.00005	M	0-3	9	1	0	0	
	Ab + HF + Si + Bo	MB	10	13.26	0.72	12.7	13.8	<0.00005	<0.00005	H	0-3	0	6	4	0	
	DB + Si + Bo	MB	10	10.62	0.59	10.2	11.0	<0.00005	0.0089	M	0-3	5	4	1	0	
Ahrari, 2013	HF + Si + Bo	MB	10	7.31	3.81	4.6	10.0	0.3052	0.0525	M						
	CO_2_-10 W-200 Hz + Si + Bo	MB	10	5.7	1.81	4.4	7.0	0.6128	<0.00005	M						
	CO_2_-15 W-200 Hz + Si + Bo	MB	10	5.2	2.8	3.2	7.2	0.3898	0.0004	M						
	CO_2_-20 W-200 Hz + Si + Bo	MB	10	4.4	2.11	2.9	5.9	0.0400	<0.00005	F						
Ramos, 2012	HF + Si + Bo	CB	10	17.5	1.56	16.4	18.6	<0.00005	<0.00005	H	0-3	0	3	5	0	
	HF + Bo	CB	10	6.1	1.66	4.9	7.3	0.8531	<0.00005	M	0-3	2	6	2	0	
	DB + PA37% + Bo	CB	10	4.8	0.68	4.3	5.3	0.0003	<0.00005	F	0-3	4	6	0	0	
Poosti, 2012	HF + Bo	MB	20	7	2.1	6.0	8.0	0.0465	<0.00005	M						
	Er:YAG-2 W + Bo	MB	20	2.3	1.1	1.8	2.8	<0.00005	<0.00005	F						
	Er:YAG-3 W + Bo	MB	20	3.7	2.3	2.6	4.8	0.0003	<0.00005	F						
	Nd:YAG-0.8 W + Bo	MB	20	6.9	2.7	5.6	8.2	0.1525	<0.00005	M						
Girish, 2012	HF + Si + Bo	MB	10	12.83	0.5645	12.4	13.2	<0.00005	<0.00005	H	0-3	2	6	2	0	
	HF + Bo	MB	10	8.707	0.3531	8.5	9.0	<0.00005	<0.00005	M	0-3	2	7	1	0	
	Ab + Bo	MB	10	7.45	0.6345	7.0	7.9	<0.00005	<0.00005	M	0-3	1	9	0	0	
	Ab + Si + Bo	MB	10	15.179	0.3844	14.9	15.5	<0.00005	<0.00005	H	0-3	0	6	4	0	
	DB + Bo	MB	10	8.396	0.7043	7.9	8.9	<0.00005	<0.00005	M	0-3	7	3	0	0	
	DB + Si + Bo	MB	10	7.764	0.739	7.2	8.3	<0.00005	<0.00005	M	0-3	8	2	0	0	
Saraç, 2011	Cojet + Si + Bo	MB	20	23.51	3.11	22.1	25.0	<0.00005	<0.00005	H	0-3	20	0	0	0	
	Ab + Si + Bo (25-micron)	MB	20	13.58	2.56	12.4	14.8	<0.00005	<0.00005	H	0-3	20	0	0	0	
Saraç, 2007	HF + Si + Bo	MB	10	5.39	2.59	3.5	7.2	0.4754	0.0003	M						
	Ab + Si + Bo (25-micron)	MB	10	17.9	3.22	15.6	20.2	<0.00005	<0.00005	H						
	Ab + HF + Si + Bo (25-micron)	MB	10	20.37	3.02	18.2	22.5	<0.00005	<0.00005	H						
Karan, 2007	Ab + Bo	MB	14	3.2	2.7	1.6	4.8	0.0019	<0.00005	F	0-3	14	0	0	0	
	Ab + Si + Bo	MB	14	10.7	5.1	7.8	13.6	0.0043	0.6162	M	0-3	2	6	2	0	
	Ab + HF + Bo	MB	14	11.3	4.1	8.9	13.7	0.0003	0.2567	M	0-3	12	2	0	0	
	Ab + HF + Si + Bo	MB	14	10.5	6	7.0	14.0	0.0149	0.7601	M	0-3	6	2	3	0	
	Cojet + Si + Bo	MB	14	15.2	5.9	11.8	18.6	<0.00005	0.0058	H	0-3	0	3	10	0	
Türkkahraman, 2006	HF + Si + Bo	C	10	11.38	1.65	10.2	12.6	<0.00005	0.0267	H						
	Ab + Si + Bo	CB	10	5.46	1.34	4.5	6.4	0.2345	<0.00005	M						
	Ab + HF + Si + Bo	CB	10	10.45	1.15	9.6	11.3	<0.00005	0.2472	M						
Türk, 2006	HF + Si + Bo	MB	10	5.39	2.59	3.5	7.2	0.4754	0.0003	M	0-3	10	0	0	0	
	Ab + Si + Bo (50-micron)	MB	10	14.66	3.17	12.4	16.9	<0.00005	0.0012	H	0-3	10	0	0	0	
	Ab + Si + Bo (25-micron)	MB	10	17.9	3.22	15.6	20.2	<0.00005	<0.00005	H	0-3	10	0	0	0	
	DB + Si + Bo (fine bur)	MB	10	26.38	3.63	23.8	29.0	<0.00005	<0.00005	H	0-3	10	0	0	0	
	DB + Si + Bo (extra-fine bur)	MB	10	24.26	4.87	20.8	27.7	<0.00005	<0.00005	H	0-3	10	0	0	0	
Akova, 2005	HF + Si + Bo	MB	10	15.07	1.44	14.0	16.1	<0.00005	<0.00005	H						
	HF + Bo	MB	10	10.78	0.62	10.3	11.2	<0.00005	0.0032	M						
	PA37% + Bo	MB	10	2.36	0.41	2.1	2.7	<0.00005	<0.00005	F						
	PA37% + Si + Bo	MB	10	10.73	1.12	9.9	11.5	<0.00005	0.0694	M						
	Ab + Bo	MB	10	2.04	0.41	1.7	2.3	<0.00005	<0.00005	F						
	Ab + Si + Bo	MB	10	13.81	2	12.4	15.2	<0.00005	0.0002	H						
	CO_2_-2 W-2 Hz + Bo	MB	10	6.26	0.58	5.8	6.7	0.1900	<0.00005	M						
	CO_2_-2 W-2 Hz + Si + Bo	MB	10	8.25	0.9	7.6	8.9	<0.00005	0.0002	M						
Tengrungsun, 2004	HF + Si + Bo	MB	16	13.25						H						
	Ab + Si + Bo	MB	16	12.41						H						
	Nd:YAG-3 W-20 Hz + Si + Bo	MB	16	11.71						M						
Özcan, 2004	HF + Bo	PC	6	11.2	2.3	8.8	13.6	0.0026	0.2574	M						
	PA37% + Bo	PC	6	8.5	2.8	5.6	11.4	0.0804	0.2465	M						
	Cojet + Si	PC	6	12	2.9	9.0	15.0	0.0039	0.1520	M						
	Ab + Si (30-micron)	PC	6	13.6	2.2	11.3	15.9	0.0004	0.0102	H						
	Ab + Si + Bo (30-micron)	PC	6	10.9	2.8	8.0	13.8	0.0078	0.4667	M						
Schmage, 2003	HF + Si + Bo	MB	10	12.2	3.4	9.8	14.6	0.0003	0.0711	M						
	HF + Bo	MB	10	14.7	3.3	12.3	17.1	<0.00005	0.0015	H						
	Ab + Bo	MB	10	2.8	1.5	1.7	3.9	<0.00005	<0.00005	F						
	Ab + Si + Bo	MB	10	15.8	4.2	12.8	18.8	<0.00005	0.0018	H						
	Cojet + Si + Bo	MB	10	14.9	3.8	12.2	17.6	<0.00005	0.0028	H						
	DB + Bo	MB	10	1.6	0.8	1.0	2.2	<0.00005	<0.00005	F						
Sant'Anna, 2002	HF + Si + Bo	MB	22	16.12	7.77	12.7	19.6	<0.00005	0.0013	H	0-3	0	1	4	5	
	Ab + Si + Bo	MB	22	18.64	7.61	15.3	22.0	<0.00005	<0.00005	H	0-3	0	0	4	7	
	DB + Si + Bo	MB	22	17.11	7.37	13.8	20.4	<0.00005	0.0002	H	0-3	1	0	2	4	
Bourke, 1999	HF + Si + Bo	MB	10	10.29	1.3	9.4	11.2	<0.00005	0.4984	M	0-3	0	4	5	1	
	HF + Bo	MB	10	3.52	0.24	3.3	3.7	<0.00005	<0.00005	F	0-3	7	3	0	0	
	PA37% + Bo	MB	10	0	0					F	0-3	10	0	0	0	
	PA37% + Si + Bo	MB	10	10.04	2.84	8.0	12.1	0.0015	0.9654	M	0-3	5	0	4	1	

^∗^ARI scores A to D, respectively, indicate 0 to 3 in the 4-score systems. ARI scores A to E, respectively, indicate 5 to 1 in the 5-score systems. Note that the 4- and 5-score ARI systems are intentionally presented in reverse orders, so that the “A” score always indicates that no adhesive remained on the porcelain. R: result; M: moderate bond strength; H: high bond strength; F: failed bond: These are determined based on statistical comparisons in most cases; for 9 groups, statistical comparisons were not technically possible and these F/M/H results were decided subjectively by comparing with similar results in other groups. Two mean bond strengths = 10.62 and =10.78 MPa were intentionally marked as “M” despite their mean SBS being statistically significantly above 10 MPa, because the significant difference from 10 was small and also since similar bond strengths from other groups were all moderate. HF: hydrofluoric acid; Si: silane; Bo: bonding; PA: phosphoric acid; AB: abrasion; DB: diamond bur; FS: femtosecond laser; Br: bracket; MB: metal bracket; CB: ceramic bracket; PC: polycarbonate.

**Table 3:** Aggregated ARI scores. Each ARI cell in each row shows the number of all specimens (in all possible studies having that particular treatment) that had that particular ARI score. For computing the first *P* value, statistical comparisons are performed between the gold standard (the first group) and the rest of groups, using the chi-squared test. The second *P* value is calculated using the chi-squared goodness-of-fit test, against an evenly distributed hypothetical target. All surface treatments have “bonding application”.

ARI system	Br	Surface treatment	*N*	ARI–number (and %) in each group					*P*_1_	*P*_2_
				A	B	C	D	E		
4-score (0 to 3)	M	HF + silane + Bo	9	21 (22.1)	29 (30.5)	27 (28.4)	18 (18.9)	—	—	0.345
		HF + Bo	6	26 (40.6)	20 (31.3)	9 (14.1)	9 (14.1)	—	0.037	0.004
		HF + Er:CrYSGG-3 W-10 Hz + Bo	1	2 (18.2)	7 (63.6)	1 (9.1)	1 (9.1)	—	0.159	0.029
		HF + Er:YAG-3 W-10 Hz + Bo	1	7 (63.6)	3 (27.3)	1 (9.1)	0	—	0.019	0.015
		PA37% + Bo	1	10 (100)	0	0	0	—	<0.0005	<0.0005
		PA37% + silane + Bo	3	20 (51.3)	11 (28.2)	7 (17.9)	1 (2.6)	—	0.003	<0.0005
		Abrasion + Bo	4	25 (53.2)	14 (29.8)	5 (10.6)	3 (6.4)	—	0.001	<0.0005
		Abrasion + silane + Bo	9	64 (49.6)	34 (26.4)	19 (14.7)	12 (9.3)	—	<0.0005	<0.0005
		Abrasion + HF + Bo	2	21 (87.5)	3 (12.5)	0	0	—	<0.0005	<0.0005
		Abrasion + HF + silane + Bo	3	6 (19.4)	11 (35.5)	10 (32.3)	4 (12.9)	—	0.833	0.238
		Cojet + silane + Bo	3	26 (54.2)	10 (20.8)	11 (22.9)	1 (2.1)	—	<0.0005	<0.0005
		DB + Bo	1	7 (70)	3 (30)	0	0	—	0.006	0.004
		DB + silane + Bo	5	35 (61.4)	13 (22.8)	5 (8.8)	4 (7)	—	<0.0005	<0.0005
		Er:YAG-2 W-10 Hz + silane + Bo	1	0	3 (30)	5 (50)	2 (20)	—	0.308	0.158
		Er:YAG-2 W-10 Hz + Bo	1	0	3 (23.1)	6 (46.2)	4 (30.8)	—	0.168	0.123
		Er:CrYSGG-3 W-10 Hz + Bo	1	10 (90.9)	1 (9.1)	0	0	—	<0.0005	<0.0005
		Er:YAG-3 W-10 Hz + Bo	1	10 (90.9)	0	1 (9.1)	0	—	<0.0005	<0.0005
		Nd:YAG-2 W-10 Hz + silane + Bo	1	0	2 (20)	6 (60)	2 (20)	—	0.143	0.055
		CO_2_-2 W-2 Hz + silane + Bo	1	7 (58.3)	4 (33.3)	1 (8.3)	0	—	0.026	0.019
	C	HF + silane + Bo	2	5 (35.7)	4 (28.6)	5 (35.7)	0	—	—	0.183
		HF + Bo	1	2 (20)	6 (60)	2 (20)	0	—	0.306	0.055
		PA37% + silane + Bo	1	6 (100)	0	0	0	—	0.030	<0.0005
		Abrasion + silane + Bo	1	4 (40)	6 (60)	0	0	—	0.083	0.013
		DB + silane + Bo	1	5 (50)	5 (50)	0	0	—	0.102	0.019
		DB + PA37% + Bo	1	4 (40)	6 (60)	0	0	—	0.083	0.013
		ND:YAG-15 Hz + silane + Bo	1	2 (25)	6 (75)	0	0	—	0.065	0.007
5-score (5 to 1)	M	HF + silane + Bo	4	7 (13)	9 (16.7)	8 (14.8)	16 (29.6)	14 (25.9)	—	0.063
		PA37% + silane + Bo	3	17 (40.5)	14 (33.3)	4 (9.5)	4 (9.5)	3 (7.1)	0.001	<0.0005
		Nd:YAG-0.75 W + silane + Bo	1	3 (25)	2 (16.7)	4 (33.3)	2 (16.7)	1 (8.3)	0.316	0.676
		Nd:YAG-1 W + silane + Bo	1	2 (16.7)	2 (16.7)	4 (33.3)	2 (16.7)	2 (16.7)	0.574	0.816
		Nd:YAG-1.25 W + silane + Bo	1	0	1 (8.3)	4 (33.3)	5 (41.7)	2 (16.7)	0.325	0.122
		Nd:YAG-1.5 W + silane + Bo	1	2 (16.7)	5 (41.7)	3 (25)	1 (8.3)	1 (8.3)	0.154	0.314
		Nd:YAG-2 W-10 Hz + silane + Bo	1	1 (8.3)	1 (8.3)	5 (41.7)	3 (25)	2 (16.7)	0.327	0.314
	C	HF + silane + Bo	2	5 (22.7)	7 (31.8)	6 (27.3)	2 (9.1)	2 (9.1)	—	0.261
		PA37% + silane + Bo	2	3 (13.6)	11 (50)	8 (36.4)	0	0	0.339	<0.0005

Br: bracket; M: metal; C: ceramic; N: number of articles; HF: hydrofluoric acid; Bo: bonding; PA: phosphoric acid; DB: diamond bur. ^∗^ARI scores A to D, respectively, indicate 0 to 3 in the 4-score systems. ARI scores A to E, respectively, indicate 5 to 1 in the 5-score systems. Note that the 4- and 5-score ARI systems are intentionally presented in reverse orders, so that the “A” score always indicates that no adhesive remained on the porcelain.

**Table 4:** The summary of the risk of bias assessment.

1st author	Reference	Country, year	1	2	3	4	5	6	7	8
Ghozy, E. A.	[23]	Egypt, 2020	?	Y	N	N	Y	Y	N	N
Kurt, I.	[30]	Turkey, 2019	?	Y	N	N	Y	Y	N	N
Mirhashemi, A.	[44]	Iran, 2018	Y	Y	N	N	Y	Y	N	N
Mehmeti, B.	[24]	Croatia, 2018	?	Y	N	N	Y	Y	N	N
Cevik, P.	[45]	Turkey, 2018	?	Y	N	N	Y	Y	N	N
Cevik, P.	[22]	Turkey, 2017	?	Y	N	N	Y	Y	N	N
Durgesh, B. H.	[36]	Saudi Arabia, 2016	Y	Y	N	N	Y	Y	N	N
Alakus Sabuncuoglu, F.	[27]	Turkey, 2016	Y	Y	N	N	Y	Y	N	N
Stella, J. P. F.	[26]	Brazil, 2015	Y	Y	N	N	Y	Y	N	N
Erdur, E. A.	[37]	Turkey, 2015	Y	Y	N	N	Y	Y	N	N
Aksakalli, S.	[46]	Turkey, 2015	Y	Y	N	N	Y	Y	N	N
Akpinar, Y. Z.	[38]	Turkey, 2015	Y	Y	N	N	Y	Y	N	N
Zarif Najafi, H.^∗^	[39]	Iran, 2014	Y	Y	N	N	Y	Y	N	N
Yassaei, S.	[50]	Iran, 2013	Y	Y	N	N	Y	Y	N	N
Purmal, K.	[28]	Malaysia, 2013	Y	Y	N	N	Y	Y	N	N
Hosseini, M. H.	[12]	Iran, 2013	Y	Y	N	N	Y	Y	N	N
Ganesan, J.	[29]	India, 2013	?	Y	N	N	Y	Y	N	N
Ahrari, F.^∗^	[62]	Iran, 2013	Y	N	N	N	Y	Y	N	N
Ramos, T.	[63]	Brazil, 2012	Y	Y	N	N	Y	Y	N	N
Poosti, M.	[14]	Iran, 2012	Y	Y	N	N	Y	Y	N	N
Girish, P. V.	[31]	India, 2012	?	Y	N	N	Y	Y	N	N
Saraç, Y. S.	[47]	Turkey, 2011	Y	Y	N	N	Y	Y	N	N
Saraç, Y. Ş.	[35]	Turkey, 2007	Y	Y	N	N	Y	Y	N	N
Karan, S.	[48]	Turkey, 2007	?	Y	N	N	Y	Y	N	N
Türkkahraman, H.	[64]	Turkey, 2006	Y	Y	N	N	Y	Y	N	N
Türk, T.	[32]	Turkey, 2006	Y	Y	N	N	Y	Y	N	N
Akova, T.	[2]	Turkey, 2005	Y	Y	N	N	Y	Y	N	N
Tengrungsun, T.	[43]	Thailand, 2004	Y	Y	N	N	Y	Y	N	N
Özcan, M.	[49]	Finland, 2004	Y	Y	N	N	Y	Y	N	N
Schmage, P.	[34]	Germany, 2003	?	Y	N	N	Y	Y	N	N
Sant'Anna, E. F.	[33]	Brazil, 2002	Y	Y	N	N	Y	Y	N	N
Bourke, B. M.^∗^	[25]	UK, 1999	?	Y	N	N	Y	Y	N	N

(1) Was there adequate randomization? (2) Were baseline conditions similar across different groups? (3) Were experimental procedures similar for different groups? (4) Were operators blinded to the grouping? (5) Were outcome data complete without missing? (6) Were all measured outcomes adequately reported? (7) Were there any reports of outcomes that were not adequately explained in methods? (8) Any other inconsistency or source of bias. Y: yes; N: no; ?: not mentioned and not obtainable. ^∗^In these studies, groups of glazed porcelain surfaces were included and deglazed porcelain surfaces were excluded.

## Data Availability

All the data and material are already presented as the manuscript, tables, and figures.
